# Physiologically Based Toxicokinetic Modeling of Bisphenols in Zebrafish (*Danio rerio*) Accounting for Variations in Metabolic Rates, Brain Distribution, and Liver Accumulation

**DOI:** 10.1021/acs.est.2c01292

**Published:** 2022-07-07

**Authors:** Ioana Chelcea, Stefan Örn, Timo Hamers, Jacco Koekkoek, Jessica Legradi, Carolina Vogs, Patrik L. Andersson

**Affiliations:** † Department of Chemistry, Umeå University, SE-901 87 Umeå, Sweden; ‡ Department of Biomedical Sciences and Veterinary Public Health, Swedish University of Agricultural Sciences, Box 7028, SE-75007 Uppsala, Sweden; § Department of Environment & Health, Vrije Universiteit Amsterdam, 1081 HV Amsterdam, The Netherlands; ∥ Institute of Environmental Medicine, Karolinska Institutet, SE-171 65 Solna, Sweden

**Keywords:** biotransformation, PBTK, zebrafish, bisphenols, endocrine disruptors

## Abstract

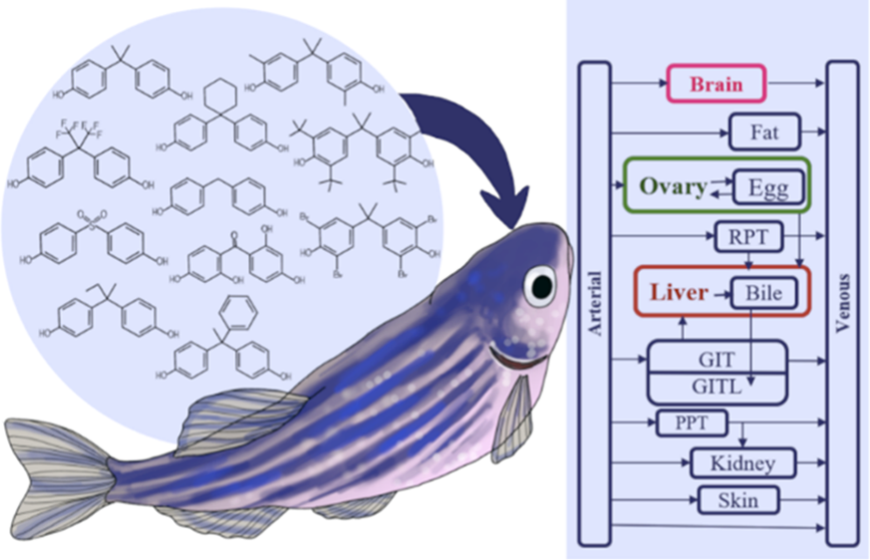

Bisphenol A (BPA) is an industrial chemical, which has raised human health and environmental concerns due to its endocrine-disrupting properties. BPA analogues are less well-studied despite their wide use in consumer products. These analogues have been detected in water and aquatic organisms around the world, with some analogues showing toxic effects in various species including fish. Here, we present novel organ-specific time-course distribution data of bisphenol Z (BPZ) in female zebrafish (*Danio rerio*), including concentrations in the ovaries, liver, and brain, a rarely sampled organ with high toxicological relevance. Furthermore, fish-specific *in vitro* biotransformation rates were determined for 11 selected bisphenols. A physiologically based toxicokinetic (PBTK) model was adapted for four of these bisphenols, which was able to predict levels in the gonads, liver, and brain as well as the whole body within a 2–5-fold error with respect to experimental data, covering several important target organs of toxicity. In particular, predicted liver concentrations improved compared to currently available PBTK models. Predicted data indicate that studied bisphenols mainly distribute to the carcass and gonads and less to the brain. Our model provides a tool to increase our understanding on the distribution and kinetics of a group of emerging pollutants.

## Abstract 2

This study models and measures the kinetic distribution of environmentally relevant bisphenols in fish organs and provides data on their metabolism.

## 1 Introduction

Endocrine-disrupting compounds (EDCs) have become a focus point in toxicology research due to their ability to interfere with the hormone systems of vertebrates.^1,2^ Estrogen-mimicking compounds can bind to and activate the estrogen receptor (ER) in various target organs, leading to downstream endocrine-disrupting effects such as development and reproduction effects.^3^ Bisphenol A (BPA) is a high-production-volume chemical with estrogen-mimicking properties.^4,5^ Its frequent use in polycarbonate plastics, thermal paper inks, and food packaging has raised increased concerns about human and animal exposures. BPA has been detected in surface waters, groundwater, effluents, and sediments^6−10^ as well as in human urine, plasma,^11−14^ and in many fish species^15^ around the world. Animal studies in rodents, fish, and reptiles have reported endocrine-disrupting effects such as feminization and disrupted spermatogenesis in males as well as reduced reproductive capacity in females and disrupted gonad development in offspring upon exposure to BPA.^4,7^

Due to the health concerns of BPA, various bisphenols are used as BPA replacements as well as for other applications such as in the production of polycarbonate plastics, in printing ink, or even in cosmetic products.^16−18^ Bisphenols such as bisphenol B (BPB), bisphenol S (BPS), bisphenol F (BPF), bisphenol Z (BPZ), or bisphenol AF (BPAF) have been detected in humans as well as in various fresh water and marine fish species.^11,15,19−21^ An emerging concern is that BPAF, BPB, and BPZ show higher bioaccumulation in fish than BPA.^22^ Some of these analogues also induce ER activation^23^ and cause similar developmental and reproductive dysfunction in fish as BPA.^3,24^

The EDC-related effects are influenced by the dose reaching the target, the rate at which it is eliminated from the body, and the intrinsic property of the compound to elucidate an effect.^25^ Toxicokinetic processes including absorption, distribution, metabolism, and elimination (ADME) are therefore of uttermost importance to estimate the bisphenol doses at targets such as the liver, brain, and gonads. Physiologically based toxicokinetic (PBTK) models can improve the understanding of the ADME properties of environmental pollutants and thus the estimation of dose at the target. PBTK models have been used to extrapolate chemical accumulation between fish species, exposure doses, and chemicals and for *in vitro*-to-*in vivo* extrapolations.^26−28^ These models represent a rapid, less costly, and ethically preferred alternative to *in vivo* experiments.^29^ However, currently, PBTK models are mainly used for refinement and support rather than a replacement for *in vivo* data. Several generic zebrafish (*Danio rerio*)^27,30^ PBTK models have been developed using validation data for neutral organic compounds including BPA.

Zebrafish is one of the most frequently utilized fish species in EDC research^31^ and is used as a model organism in both environmental and human toxicology,^32−34^ and understanding the toxicokinetics of organic chemicals in this organism could facilitate extrapolation between species. However, existing PBTK models for zebrafish have not been validated for bisphenols using time-course organ-specific concentrations, which is warranted to understand the toxicokinetic and endocrine properties of bisphenol analogues.^27,30^

The present study aims at advancing currently available PBTK models for multiple bisphenols by enlarging the chemical domain and improving predictions at the tissue level. BPZ was chosen for *in vivo* kinetic studies as it has been detected in human urine^14^ and serum^12^ samples, food,^35^ personal care products,^17^ and various environmental matrixes such as sludge^36^ and sediment.^37^ Furthermore, BPZ has shown considerably higher bioaccumulation than BPA in fish.^22^ Notably, data on metabolization of BPA analogues in fish are currently missing, despite this being considered the main route of eliminating parent compounds *in vivo*(38,39) and, therefore, one of the most important parameters for understanding toxicokinetics in fish.^40^ We aimed to address this data limitation by measuring *in vitro* liver metabolism for the selected bisphenols to accurately parametrize the main route of elimination. We refined and extended the existing PBTK model using both literature and our own experimental data. Finally, PBTK models were used to predict bioconcentration potential in fish organs for the selected bisphenols.

## 2 Materials and Methods

The molecular structures and the process for selecting an environmentally relevant subset of bisphenols are described in Section 1. The selection process considered exposure risk for humans and aquatic organisms, environmental levels, and predicted estrogenic activity. The selected bisphenols (Figure S1) and information about their use and estrogenic properties are provided in Table S1.

### 2.1 Chemicals

BPA (CAS 80-05-7), BPF (CAS 620-92-8), BPS (CAS 80-09-1), BPAF (CAS 1478-61-1), benzophenone 2 (BP-2) (CAS 131-55-5), BPZ (CAS 843-55-0), bisphenol AP (BPAP) (CAS 1571-75-1), tetrabromobisphenol A (TBBPA) (CAS 79-94-7), Bimox M (CAS 118-82-1), bisphenol C (BPC) (CAS 79-97-0), and BPB (CAS 77-40-7) were purchased from Sigma-Aldrich in crystal form with 99% purity for all experiments.

### 2.2 Biotransformation Rate Estimation

*In vitro* metabolic rates of selected bisphenols were determined using rainbow trout liver homogenate according to OECD TG 319.^41^ Duplicate incubations of the individual bisphenols were performed on two separate days. In brief, rainbow trout liver S9 homogenate was preincubated in buffer and cofactors, followed by 120 min of incubation with bisphenol. From each duplicate, 50 μL of subsamples was taken at timepoints 0, 2, 5, 15, 30, 60, 90, and 120 min and mixed with 200 μL of ice-cold methanol. The protocol of the procedure is described further in Section 2. Time-dependent depletion of parent bisphenols in the metabolic rate mixtures was measured by liquid chromatography-tandem mass spectrometry (LC-MS/MS) according to the protocol described in Section S4.1 and Tables S2–S4.

Disappearance rates of the parent bisphenols were determined by log-linear regression as the first-order elimination rate constant for each incubation separately. All of the timepoints up to 120 min were included in the regression, except for those bisphenols reaching the analytical limit of detection within the incubation period, *i.e.*, BPB (maximum incubation time of 90 min), BPAP (60 min), and BP-2 (5 min). First-order elimination rate constants (average of two replicate experiments) were calculated into intrinsic clearance rates as suggested by the OECD,^41^ which were again transformed for model parameterization assuming an S9 protein concentration of 163 mg/g liver.^42^ Final values used for model parameterization are shown in Table 1.

**Table 1:** Selected Environmentally Relevant Bisphenols, Their Corresponding Chemical Properties Used for PBTK Model Parameterization, and Predicted Bioconcentration Factor (BCF) Values

					BCF^e^			
name	log* K*_ow_^a^	CL^b^ (mL/d/g liver)	*P*_bw_^c^	*P*_livb_^d^	whole body	liver	gonad	brain
BPA	3.42^f^	2.45 × 10^3^	1.45	195^g^	17	120	13	1.9
BPAF	3.74	3.04 × 10^3^	0.71	70.0^g^	7.8	18	6.9	1.0
BPAP	4.38	7.06 × 10^3^	4.15	3.08	40	1.9	37	5.2
BPB	3.94	3.76 × 10^3^	1.78	3.05	18	1.6	17	2.4
BPC	4.34	4.04 × 10^3^	3.88	3.08	39	3.2	36	5.1
BPF	2.91^h^	1.20 × 10^3^	1.40	3.02	13	3.1	12	1.8
BPS	1.73	1.77 × 10^3^	0.14	3.19	1.4	0.2	1.3	0.2
BPZ	4.34	1.16 × 10^3^	3.88	3.08	46	11	43	6.1
BP-2	2.69	5.79 × 10^4^	0.42	3.02	5.8	4.0 × 10^–3^	5.4	0.8
TBBPA	6.53^i^	1.63 × 10^4^	13.1	3.24	79	1.7	75	10
Bimox M	9.06	0	5.42 × 10^3 ^j	3.43	1.3 × 10^4^	1.4 × 10^4^	1.3 × 10^4^	1.7 × 10^3^

a Median prediction of log *K*_ow_ from the CompTox Dashboard.

b Clearance rate determined in the present study by *in vitro* incubation with rainbow trout liver S9.

c Blood–water partitioning predicted with the model by Fitzsimmons et al.^49^

d Liver-to-blood partitioning predicted with the model by Bertelsen et al.^50^

e Bioconcentration factors predicted using the PBTK model developed in the present study.

f Staples et al.^67^

g Fitted in the current study based on experimental data.

h Measured value from the CompTox Dashboard.

i Measured value by Kuramochi et al.^68^

j Out of the log *K*_ow_ range of the *P*_bw_ model domain thus likely to be inaccurate.

### 2.3 *In Vivo* Zebrafish Kinetics of BPZ

Female zebrafish were exposed to a nominal water concentration of 10 μg/L BPZ for 14 days, followed by a 6-day depuration phase. At sampling, adult fish were euthanized by immersion in a sodium bicarbonate-buffered tricaine methanesulfonate solution (MS222: 500 mg/L) and decapitated thereafter. The liver, ovaries, brain, and carcass from three fish were sampled at 6, 12, 24, 48, 72, 336, 411, and 480 h of exposure as well as at 3, 6, 12, 24, and 144 h after the start of the depuration phase. Additionally, three whole fish were sampled at timepoints of 6, 12, and 24 h of the exposure phase. Water from the fish tank was sampled at 0, 12, 24, 48, 72, 168, 336, 411, and 480 h after exposure start as well as at 0, 12, 24, and 96 h after the start of depuration. Control fish and tank water were sampled before the start of exposure. Details of fish husbandry can be found in Section 3, and a schematic of the experimental setup is presented in Figure S2. All samples were immediately stored at −20 °C until further analysis. The analysis of BPZ in zebrafish and water samples was carried out by isotope dilution and LC-MS/MS, as described in Sections S4.2 and S4.3 and Tables S5–S8.

### 2.4 Physiologically Based Toxicokinetic Modeling of Adult Zebrafish

#### 2.4.1 Experimental Data Collection

For PBTK model development, we included published quantified concentrations in either the whole body or organs of adult or juvenile zebrafish for any of the selected bisphenols. Zebrafish-specific *in vivo* data for BPA was obtained from Lindholst et al.,^39^ Chen et al.,^43^ and Fang et al.^44^ Data from Fang et al.^44^ were considered uncertain due to large variation in measured BPA water concentrations of compound, which was likely caused by semistatic exposure design. This study was therefore not included in model calibration and validation but is still presented and discussed for comparison. Lindholst et al.^39^ also provided kinetic data on the glucuronic acid conjugate, which was used to calibrate the modeling of this BPA metabolite. Experimental data for BPAF and its glucuronic acid conjugate were collected from Shi et al.^45^ TBBPA data were obtained from Nyholm et al.^46^ We only identified organ-specific zebrafish data for BPA and BPAF in the liver, brain, and gonads.^43,44,47^ Data extraction from graphs was performed using WebPlotDigitizer^48^ when numeric values were not provided by the authors. An overview of all experimental data used is listed in Table S9.

#### 2.4.2 Model Structure

**Figure 1:**
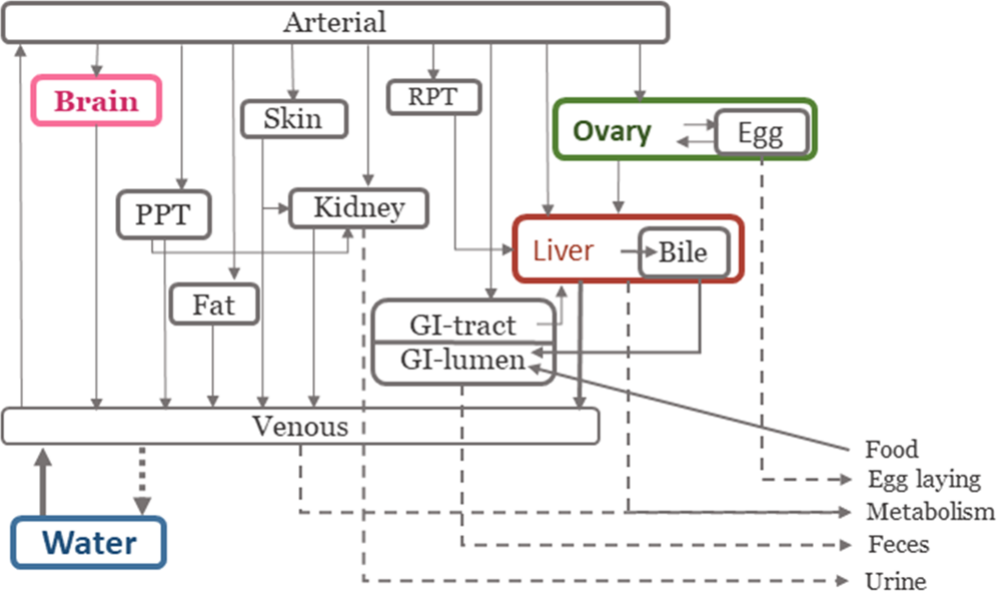
PBTK model structure for adult female zebrafish adopted from Grech et al.^27^ Solid arrows represent mass balance flows between organs, and dashed arrows represent possible elimination pathways. Colored compartments represent organs for which experimental data were available. Abbreviated compartment names are richly perfused tissue (RPT), poorly perfused tissue (PPT), and gastrointestinal (GI) tract/lumen.

A zebrafish PBTK model with 13 compartments was developed further based on a 12-compartment model by Grech et al.^27^ with structural and parameter alterations described below in Figure 1, illustrating the structure of the female PBTK model, while Figures S3 and S4 show the structure for male and metabolite models, respectively. The model was advanced using data for BPA, BPAF, BPZ, and TBBPA as well as two metabolites, BPA glucuronic acid (BPA-GA) and BPAF glucuronic acid (BPAF-GA). A 13th compartment, namely, eggs, was added within the ovary to model the maternal transfer of bisphenols to offspring. The whole-body concentrations were calculated using the sum of chemical amounts in all organs divided by the total bodyweight. Absorption occurred either through food ingestion *via* the gastrointestinal lumen (GIL) or through gill respiration. Gill absorption of bisphenols from water was modeled directly into the venous compartment. Elimination was simulated either as gill excretion into the water directly from the venous compartment or *via* urine, feces, liver metabolism, and through egg-laying for females. The PBTK model also contains two dynamic submodels, one for temperature (*T*) and one for growth described in detail in Section S5.1. The rate of chemical mass change in each compartment was modeled as amounts (μg).

#### 2.4.3 Parameters

Physiological parameters were obtained from Grech et al.^27^ and Péry et al.^30^ with minor modifications from the *in vivo* experiment performed in this study and are shown in Tables S10–S12. Chemical parameters used for model parameterization are provided in Tables 1 and S13. Additionally, metabolic rates for each bisphenol in the liver were parametrized using the *in vitro* biotransformation rates obtained for rainbow trout liver S9 in the present study (Table 1).

#### 2.4.4 Partitioning

Blood–water partition coefficients (*P*_bw_) were predicted using a quantitative structure–property relationship (QSPR) eq 1 by Fitzsimmons et al.^49^ as suggested in previous fish PBTK studies.^27,30^ The QSPR model, validated on chemicals with a log *K*_ow_ ranging from 0 to 8 (Table 1), was adjusted for the unbound fraction of compound (*F*_unbound_) (Table S13)
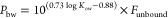
1

If experimental organ concentrations were not available, the tissue–blood partition coefficients (*P*_t_) were predicted using a QSPR model by Bertelsen et al.^50^ as follows
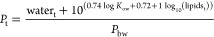
2where water_t_ and lipids_t_ represent the water and the lipid content of the tissue, respectively. Equation 2 was applied for all modeled organs and bisphenols with the exception of fitted parameters described below.

Time-course kinetic data in the brain were not available for bisphenols; therefore, we used our measured *in vivo* brain concentrations for BPZ to fit the brain–blood partition coefficient (*P*_bb_). This value was then used to parameterize all bisphenols. Liver–blood partitioning (*P*_livb_) was fitted for BPA^43^ and BPAF,^47^ since enough datasets for both fitting and validation were available for these compounds.

Toxicokinetics of the main metabolites of BPA and BPAF, namely, BPA-GA^39^ and BPAF-GA,^47^ were also modeled using existing *in vivo* data (Table S9) and parameters (Tables S13 and S14). These were the major metabolites for which measured concentrations were available from the literature. Data on BPA sulfate were also available, but concentrations were 1000 times lower than those of the glucuronide and therefore considered less relevant.^39^ The modeling and fitting approaches for metabolites are described in Section S5.2.

#### 2.4.5 Maternal Transfer in Female Zebrafish

The amount of chemicals in eggs was modeled by describing the eggs as a subcompartment of the ovaries. The maternal transfer depended on the amount of compound partitioning into the ovaries based on a diffusion-limited equation that required the permeability surface area product (PS) as described by Thompson and Beard.^51^ The subcompartment was set to start at the volume of one egg (2.12 × 10^–4^ mL), and the clutch was modeled to grow linearly, reaching the volume of an average zebrafish clutch at spawning (0.04 mL), which was assumed to occur at regular intervals of 1.5 days.^52^ After spawning, the volume of the egg compartment was returned to one egg, and the process was set to repeat indefinitely for a female zebrafish, resulting in parts of the compound being eliminated *via* the eggs. Parameters used for describing this subcompartment are shown in Table S12. The PS was calculated as a dynamic process that depended on the volume and surface area of the egg subcompartment as follows
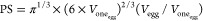
3where *V*_one_egg_ is the volume in mL of a single egg and *V*_egg_ is the volume of the growing clutch; thus, PS increases as the number of eggs increases. Eggs were assumed to be spheric.

Ovaries and eggs were assumed to receive the same blood flow. The equation for the egg compartment was described as follows using the diffusion-limited equation previously described by Thompson and Beard^51^
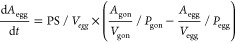
4where d*A*_egg_/d*t* represents the rate of change of bisphenol over time in the egg compartment, *A*_gon_ and *A*_egg_ represent the amount of compound in the gonads and egg, respectively, *V*_gon_ and *V*_egg_ are the volumes of the gonads and eggs, respectively, and *P*_gon_ and *P*_egg_ are the partition coefficients of the gonads to blood and egg to gonads. *P*_egg_ was fitted based on egg concentration data of TBBPA from Nyholm et al.^46^ using the high-dose dataset. This value was then used for all of the bisphenols in the list.

#### 2.4.6 Model Fit and Sensitivity Analysis

Parameter fitting was done in R using the modFit function in the FME package with the Nelder–Mead algorithm.^53^ Details on fitting methodology and data used for fitting are presented in Section S5.4 and Table S15.

The model was adjusted based on the experimental setup such as dosing regime, water temperature, bodyweight, and sex of fish from the various studies (Table S9) and for chemical-specific parameters of the different bisphenols. Predicted concentrations for whole fish or individual organs were then compared with measured data in terms of area under the curve (AUC) (μg/g/day), maximal concentration (*C*_max_) (μg/g), half-life (*t*_1/2_) (days), and bioconcentration factor (BCF). The goodness of fit was assessed by calculating the normalized root mean squared error (NRMSE)^54^ values for predicted *versus* observed data for all organs combined. NRMSE for each compound prediction was calculated by normalizing the RMSE to the maximal predicted concentration for each organ. Global sensitivity analysis was performed for BPA and BPAF models using a Sobol test,^55^ to assess the relative importance of parameters. Analysis was done for the liver and whole-body AUC both using the QSPR model for partition coefficients and using the actual values obtained from the model. Sobol sensitivity analysis was performed by varying all parameters within a uniform distribution using a variation of ±20% of their mean values. The top 10 most influential parameters for each output are shown in Figures S6 and S7.

#### 2.4.7 Software

PBTK modeling scripts were written in R (v 4.0.0) and incorporated into a KNIME (v 4.1.4)^56^ workflow. Method lsoda of the function ode in package deSolve^57^ was used for solving the differential equations. The httk package^58^ was used for calculating AUC based on noncompartmental analysis approach, while *t*_1/2_ was calculated using one-compartment linear regression. Sobol sensitivity analysis was performed using the soboljansen function of the package sensitivity (v 1.24.0). Chemical properties were predicted using EPISUITE^59^ for *W*_sol_ and using the median predictions from the CompTox Dashboard^60^ for log octanol–water partitioning (log *K*_ow_).

## 3 Results and Discussion

### 3.1 Biotransformation Rate Measurements

Biotransformation rates were determined *in vitro* for the selected bisphenols, and these varied less than an order of magnitude, with the exception of BP-2, TBBPA, and Bimox M (Table 1). Both BP-2 and TBBPA are weak acids with electron-withdrawing substituents on the phenyl rings, such as hydroxy groups and bromines, respectively, and both show quicker metabolic transformation as opposed to the other bisphenols. Notably, in both repetitions of the experiment with TBBPA, the levels stopped decreasing after 15 min of incubation when ∼70% of the parent compound had been metabolized. Therefore, the biotransformation rate used for model parameterization is based on the rate within the first 15 min. A possible explanation for this effect is enzyme inhibition by metabolites, but further studies are required to confirm this. Bimox M, on the other hand, showed no metabolic degradation within 2 h of incubation, which may be due to the larger nonpolar substituents on the rings, providing steric hindrance or due to its low water solubility and high hydrophobicity, possibly causing precipitation or adsorption on surfaces (Tables 1 and S12). Rates for replicates and a summary of the compounds where metabolism stopped before 120 min can be found in Table S16.

Biotransformation is generally considered a crucial parameter for fish PBTK modeling^40^ as well as for assessing bioaccumulation potential,^61^ but such information is often only available for humans or rats, which may differ from fish. Our sensitivity analysis showed liver metabolic clearance to be a crucial parameter both for predicting whole-body as well as liver AUC (Figures S6 and S7). When comparing human metabolic rates obtained from the CompTox Dashboard^60^ (Table S17) with those measured in the current study, we observed 1 order of magnitude lower metabolic transformation rates for six of the studied bisphenols in fish compared to humans, while BPZ, BP-2, and TBBPA showed similar rates. Our findings indicate that human metabolic rates may differ from those of fish and therefore cannot be used to accurately model fish toxicokinetics. Literature data on fish metabolism for bisphenols was only available for BPA, BPS, and BP-2,^39,62,63^ with BPA data only available as rates of a specific glucuronosyltransferase (UGT)^64^ and a specific sulfonyltransferase.^63^ Sulfation represents a minor fraction of the metabolism *in vivo,*(39) with glucuronidation being the main metabolic pathway in both fish and mammals.^39,62^ In the case of UGT1A1, Wang et al.^64^ reported an activity of 5.19 pmol/min/mg protein, which is much lower than our reported values. However, this is a single isolated enzyme, and phase I reactions may be involved in accelerating the parent compound to glucuronide metabolism. Literature data from *in vivo* and primary hepatocyte studies suggest that phase II metabolization is the main route of biotransformation for BPA, BPAF, BPS, and BP-2.^39,47,62,65^ Here, experimental studies were conducted using rainbow trout S9 to parameterize liver intrinsic clearance rate (CL_int_) for the PBTK model that accounted for both phase I and phase II metabolism by adding cofactors for both processes. Thus, the design of the study does not allow us to distinguish whether the observed metabolic rates were due to predominantly phase I, phase II, or both processes.

We applied a rainbow trout liver S9 fraction-based assay as it is commercially available, and rainbow trout is the species of choice in the OECD technical guideline 319B for *in vitro* studies on fish hepatic biotransformation rates.^41^ However, some uncertainty exists on variation in kinetics between zebrafish and rainbow trout. For example, Lindholst et al.^39^ suggested that the metabolism of BPA in zebrafish is faster than that in rainbow trout. These conclusions were based on simulations from a two-compartment elimination model fitted to their experimental observations. Experimental data from that study showed, however, that BPA levels dropped below the limit of quantification after 168 h in rainbow trout organs, while it was still detectable in the whole body of zebrafish.^39,66^ This observation indicates that the metabolic rate may be lower in zebrafish than in rainbow trout, but no definitive conclusion can be drawn as the comparison is not between the whole fish of both species. Nonetheless, our measured data are promising for future modeling of rainbow trout. We have therefore provided the estimated *in vivo* intrinsic clearance (CL_*in vivo*,int_) calculated as suggested in the OECD guideline 319B^41^ (Table S17).

### 3.2 BPZ *In Vivo* Experiment

**Figure 2:**
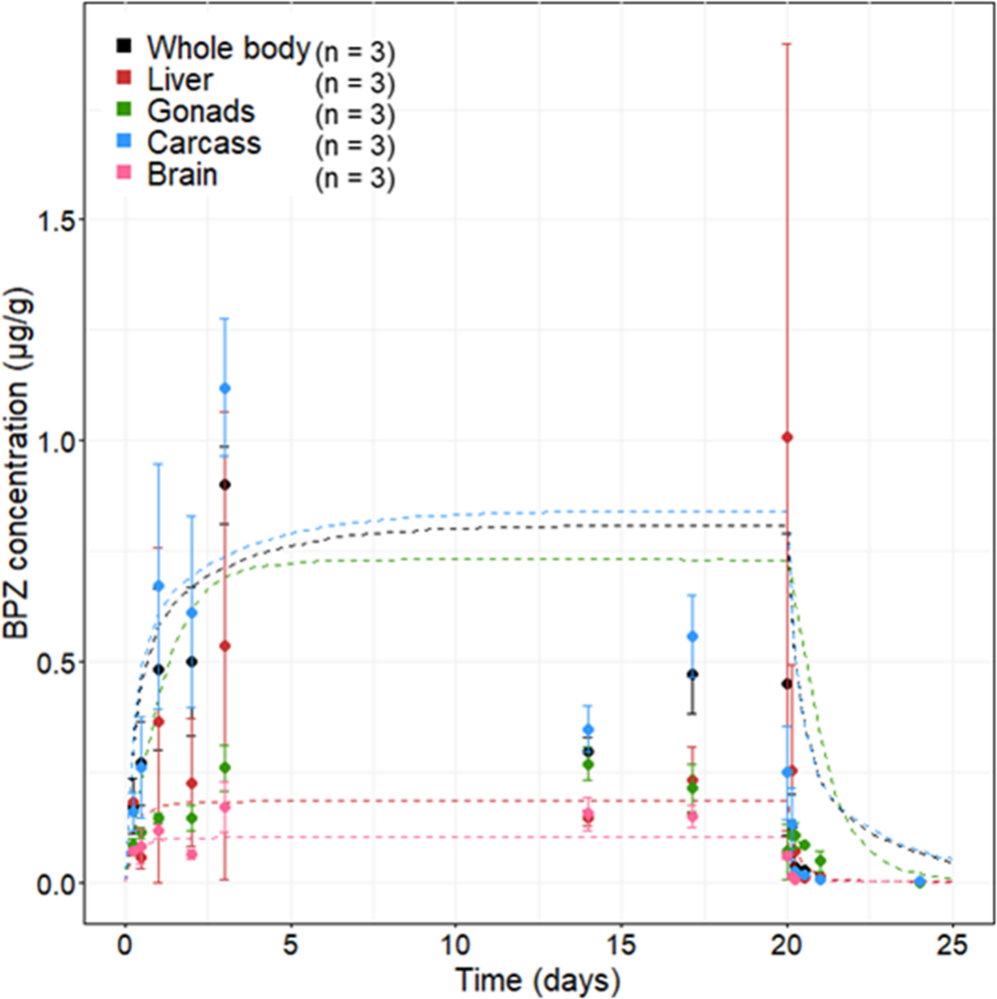
Measured internal BPZ concentrations in the liver, ovaries, brain, carcass, and whole body in female zebrafish exposed to 17 μg/L BPZ in water for 20 days with a 4-day depuration period. Dots represent measured data with error bars showing standard deviation and dotted lines represent PBTK model prediction. Observed whole-body concentrations past 24 h are calculated based on the sum of compounds in the carcass and organs and their corresponding fractions of bodyweight (measured for each individual fish).

**Figure 3:**
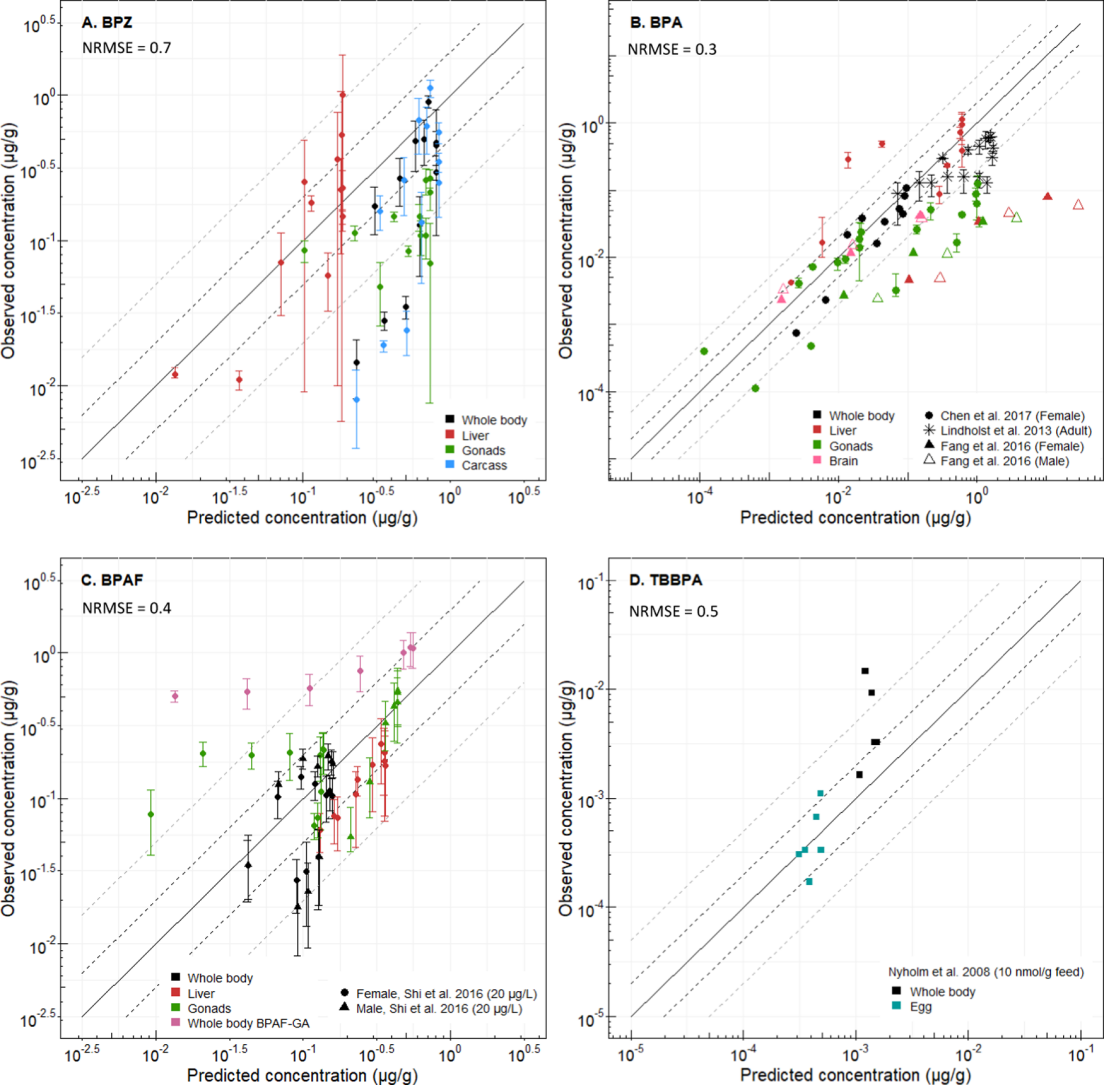
Measured *versus* predicted organ and whole-body concentrations (μg compound/g fish) of (A) BPZ, (B) BPA, (C) BPAF and BPAF-GA, and (D) TBBPA in zebrafish. Experimental data for BPA were obtained from Lindholst et al.^39^ (*n* = 4), Chen et al.^43^ (*n* = 3 of 5 pooled fish each), and Fang et al.^44^ (*n* = 3 of 5 pooled fish each), for BPAF and BPAF-GA from Shi et al.^47^ (*n* = 4), and for TBBPA from Nyholm et al.^46^ (*n* = 1 of 2 pooled individuals). The solid line represents 1:1 correlation and dotted lines represent the 5-fold (gray) and 2-fold (black) errors. NRMSE was calculated for all organs combined without the inclusion of metabolites or data used for fitting. Data used for fitting parameters were not included in the graphs.

Time-course concentrations of BPZ were determined in the carcass, liver, ovaries, brain, and whole body (Figures 2, 3, and Table S18). BPZ was not detected in control fish or control tank water as well as in depuration water. The measured mean BPZ water concentration over the exposure period was 17 ± 4.7 μg/L (Table S19); thus, the concentration of 17 μg/L was used as the PBTK model input. BPZ levels of whole-body homogenates taken during the first three exposure timepoints did not differ significantly from whole-body concentrations (*p* > 0.05 using a two-sample *t*-test for each measured timepoint) calculated using the sum of carcass, liver, ovaries, and brain adjusted for their bodyweight fraction (Table S18). This indicates that summed organ concentrations can be used as a representation of whole-body concentrations to compare with studies for other bisphenols. With the exception of the liver, measured BPZ levels in organs showed low deviation between replicates with a coefficient of variation below 50% (Table S18).

Liver concentrations varied across replicates with a coefficient of variation close to 100% for five of the measured timepoints. This is, however, not surprising as previous zebrafish *in vivo* studies also show large variations specifically in the liver compared with other organs.^43,47^ One explanation could be the large interindividual variations in CYP1A activity^69−71^ and in the gene expression of phase I and phase II enzymes^72^ that have been observed between control zebrafish. Another explanation for the larger variation in liver concentrations as compared to previous studies on BPA^43^ and BPAF^47^ can be attributed to the fact that the current study did not pool samples from several fish, and thus individual variation will be seen to a larger extent. BPZ showed higher accumulation in zebrafish than BPA and BPAF, demonstrated by BCF values for BPZ ranging from 52.9 (whole body) to 65.9 (carcass) as opposed to observed whole-body BCF values for BPA of 6.46–19.2 and for BPAF of 7.04–9.80 (Table 2). Notably, the accumulation of BPZ in the liver was lower than that of BPA but higher than that of BPAF (Table 2). To the best of our knowledge, this is the first zebrafish study investigating time-course concentrations of bisphenol A in the brain as opposed to single-value measurements. As seen in Figure 2, measured brain concentrations are consistently, although not significantly, lower than those in other organs despite the high blood flow and fat content of the organ, which could be due to the blood–brain barrier preventing the compound from entering the brain.

**Table 2:** Toxicokinetic Data in Terms of Maximal Concentration (*C*_max_), Area under the Curve (AUC), Half-Life (*t*_1/2_), and Bioconcentration Factor (BCF) for Whole-Body and Liver Concentration of BPZ, BPA, BPAF, and TBBPA

study (dose)	gender	organ	*C*_max_	*t*_1/2_	AUC	BCF	
BPZ							
current study (17 μg/L)	female	whole body	0.88	1.29	16.0	51.8	predicted
			0.9	5.58	10.6	52.9	observed
		liver	0.12	0.75	3.70	11.0	predicted
			1.01	8.75	7.07	59.2	observed
		ovaries	0.73	0.78	14.6	43.1	predicted
			0.27	10.3	4.70	15.8	observed
		brain^a^	0.10	0.76	2.06	6.07	predicted
			0.17	5.61	2.85	10.0	observed
		carcass	0.84	1.34	16.7	49.5	predicted
			1.12	4.94	12.5	65.9	observed
BPA							
Lindholst et al.^39^ (97.5 μg/L)	not specified	whole body	1.70	1.54	12.4	17.5	predicted
			0.63	4.47	4.13	6.46	observed
Chen et al.^43^ (5.72 μg/L)	female	whole body	0.10	1.76	0.59	17.5	predicted
			0.11	2.76	0.56	19.2	observed
		liver	0.61	1.25	3.68	107	predicted
			1.14	2.68	6.31	199	observed
		ovaries	0.07	0.94	0.41	12.2	predicted
			0.12	2.37	0.62	21.0	observed
Chen et al.^43^ (1.94 μg/L)	female	liver^a^	0.21	1.25	1.25	108	predicted
			0.39	1.21	1.78	201	observed
		ovaries	0.03	0.94	0.14	15.5	predicted
			0.02	2.32	0.11	10.3	observed
BPAF							
Shi et al.^47^ (20 μg/L)	male	whole body	0.16	0.93	1.10	7.97	predicted
			0.20	3.58	1.25	9.80	observed
	female	whole body	0.16	1.00	1.08	7.85	predicted
			0.14	4.99	0.8	7.04	observed
	male	liver^a^	1.06	0.50	7.41	52.9	predicted
			0.81	25.4	4.93	40.5	observed
	female	liver	0.36	0.51	2.51	18.0	predicted
			0.24	8.52	1.38	11.9	observed
	male	testes	0.44	0.49	3.08	22.0	predicted
			0.55	0.85	3.23	27.5	observed
	female	ovaries	0.12	0.70	0.80	6.14	predicted
			0.22	7.92	1.50	10.9	observed
TBBPA							
Nyholm et al.^46^ (10 nmol/g feed)	female	whole body	0.015^b^	NC^c^	0.233	NC^c^	predicted
			0.015	NC^c^	0.291	NC^c^	observed
		egg	0.001	NC^c^	0.018	NC^c^	predicted
			0.001	NC^c^	0.017	NC^c^	observed
Nyholm et al.^46^ (100 nmol/g feed)	female	egg^a^	0.005	NC^c^	0.183	NC^c^	predicted
			0.004	NC^c^	0.118	NC^c^	observed

a Data used for parameter fitting.

b *C*_max_ over the whole simulation period. Due to the dosing and sampling regime of the study, the predicted concentrations at the sampling timepoint were much lower than those right after feeding, resulting in an accurate prediction of *C*_max_ but underprediction of concentrations (Figure 3).

c Not calculated. The TBBPA study did not include a depuration phase, and the internal concentrations did not reach a steady state due to the oral dosing regime, meaning no BCF or *t*_1/2_ could be calculated.

### 3.3 Physiologically Based Toxicokinetic Modeling of Adult Zebrafish

#### 3.3.1 Whole Body

The developed PBTK model predicted the highest whole-body and carcass concentrations of BPZ, BPAF, and BPA, within a 5-fold error, and half the data points were predicted within a 2-fold error (Figure 3). Furthermore, BCF, AUC, and *C*_max_ values for whole body and carcass of BPA data from Chen et al.^43^ as well as from all data for BPAF and BPZ were predicted within a 2-fold difference from the observed data (Table 2). The model predicted 84% of whole-body BPA data with a 5-fold error for measured dosing scenarios ranging from 5.72^43^ to 97.5 μg/L.^39^ The model was also capable of predicting metabolites as AUC, and the *C*_max_ values of BPA-GA and BPAF-GA were accurately predicted within a 2-fold error (Table S20 and Section S8), although BPA-GA data were used for fitting and thus could not be validated. There was a tendency to overpredict levels of the parent compound in the case of the high-dosing scenarios (Table 2), which could be due to toxic effects, saturation of uptake, or saturated elimination processes. Nonetheless, lower doses are more relevant for environmental risk assessment as they represent more realistic exposure scenarios.

To the best of our knowledge, gender-specific variations of bisphenol accumulation in zebrafish have only been reported for BPA^44^ and BPAF.^47^ In the case of BPA, accumulation was to a similar extent in both male and female organs, whereas for BPAF, whole-body, gonad, and liver concentrations were lower in females than those in males.^47^ The model, however, predicts similar concentrations in both genders (Figure 3) despite the incorporation of an additional elimination route *via* egg-laying. Possibly the model underestimates the extent of elimination *via* eggs, which would require further validation data to confirm. Another explanation for the difference might be that the metabolic clearance capacity of the genders differs and should be parameterized gender-specific. This is supported by the fact that the concentration of the main metabolite, BPAF-GA, was reported to be nearly twice as high in females than in males, indicating a higher metabolic rate in the liver or additional metabolic capacity of nonhepatic tissues in females.^47^ Although gender-specific differences in phase II metabolism have not been thoroughly investigated, gender-specific differences in metabolic response upon chemical exposures have been previously observed in zebrafish.^73,74^

All bisphenols for which measurements were available had a consistently underpredicted half-life time (*t*_1/2_) and therefore an overpredicted AUC (Table 2). It is possible that the excretion of the parent compound *via* feces, bile, urine, or skin also plays a role in the elimination of bisphenols. These elimination routes have not yet been parameterized in the model, unlike metabolism and gill respiration. Additionally, metabolization in extrahepatic tissues has not been parameterized in the PBTK model due to a lack of experimental data, but the gill and gut have been demonstrated to be important sites of biotransformation in fish.^75^ However, hepatic metabolism seems to be the main route of eliminating internal parent bisphenol in fish as observed *in vivo*.^39^ This further highlights the need for a better understanding of the elimination of bisphenols in zebrafish.

TBBPA whole-body concentrations were on average underpredicted (Figure 3). It is, however, important to note that the *in vivo* study on TBBPA was performed using food exposure,^46^ which introduces uncertainty into the model. Apart from the variability that is expected in the feeding habits of individual fish, an important source of uncertainty is the lack of data on oral absorption of bisphenols in zebrafish, which cannot be allometrically scaled between different fish species unlike water absorption, making it difficult to model or extrapolate.^40^ Oral absorption has been previously modeled accurately in zebrafish for various compounds but with larger *in vivo* data availability.^76,77^ We have fitted the oral absorption based on egg concentrations measured at a different exposure as there were not several datasets available for whole-body concentrations. Additionally, there may be some uncertainty in the *in vitro* measurement of TBBPA clearance as described earlier in this paper.

#### 3.3.2 Liver

The AUC as well as 53% of BPZ liver measurements were predicted within a 2-fold difference of the measured values. Excluding the data used for fitting liver partitioning, 92% of liver concentrations for BPA and 100% for BPAF were predicted within a 5-fold error. Liver AUC, *C*_max_, and BCF values for these compounds were predicted within less than a 2-fold difference (Figure 3).

The previously developed zebrafish models^27,30^ have not been validated using time-course, organ-specific data for bisphenols. These models underpredict liver concentrations as suggested by recent *in vivo* studies on BPA^43^ and BPAF^47^ as well as data from other fish species.^22,66,78,79^ The model by Grech et al.^27^ was parameterized with a lower liver partitioning and higher liver clearance, resulting in very low parent compound concentrations predicted in the liver (Figure S5). To address this, we chose to refit liver partitioning and model liver concentrations as the sum of compounds in the liver and bile. This modeling approach greatly improved the liver concentration predictions (Figures 3 and S5). Biliary accumulation of the parent compound was included as an additional accumulation compartment within the liver but was not parametrized due to a lack of data. Notably, the previous model^27^ was validated using data by Fang et al.,^44^ which shows a lower degree of liver accumulation than the Chen et al.^43^ study. These two studies used the same exposure concentrations of 2 μg/L; however, the measured liver concentrations differed by up to 100-fold. Chen et al.^43^ applied a study design aligned with that proposed in the OECD guidelines;^80^ however, BPA was dosed in a mixture of various chemicals, which could influence the ADME properties. Additionally, Chen et al.^43^ monitored both water and internal concentrations throughout the dosing and depuration phases, thus providing time-course data. The Fang et al.^44^ study provides data on more organs and for both genders, but water concentrations vary throughout the exposure by up to 87% and only a single timepoint was measured at the end of exposure, thus not allowing us to investigate time-course variation in concentrations. In the current study, data from Fang et al.^44^ study were therefore only used for comparison, in particular considering brain levels, which are not reported elsewhere for these chemicals.

Generally, liver concentrations were underpredicted even with the current model, suggesting there may be another partition-independent process, such as active transport, affecting liver concentrations. A hypothesis could be that some of the parent compound can be found in the bile since biliary ducts and liver are generally analyzed within the same sample for small species such as zebrafish. Although bile accumulation of parent compounds is only a hypothesis, there is evidence supporting this assumption. Lv et al.^81^ reported that concentrations of unconjugated BPA measured in the liver and bile of wild fish were not significantly different from concentrations in plasma or muscle. The trend of higher or similar liver concentrations compared to whole-body homogenate or muscle concentrations was observed for both BPA and BPAF in zebrafish by Chen et al.^43^ and Shi et al.^47^ as well as for several bisphenols in various Atlantic ocean fish,^20^ flounder,^78^ rainbow trout,^66^ carp,^22^ and false clown anemonefish.^82^ In addition to bile, another explanation for the higher liver concentrations observed than those previously predicted could be the deconjugation of metabolites in the gut^83,84^ and reabsorption into the liver *via* the portal vein. However, this has only been described in mammals, and it is still unclear whether this process occurs in fish. To unravel these mechanisms in fish, experimental studies on gut enzymes and enterohepatic recirculation are required.

#### 3.3.3 Gonads and Eggs

Predicted gonad concentrations agreed well with experimental data for BPA and BPAF with 75 and 65% of data being predicted within a 2-fold error, respectively (Figure 3). BPAF gonad concentrations are well predicted for both genders, with predictions for males being more accurate than those for females. This can be explained by the much larger variation in female gonads over time due to the short and repeating spawning cycle. The ovary concentrations for BPZ were, however, consistently overpredicted with 63% of data points being within a 5-fold error. We sampled the ovaries of females in reproductive stages, and thus the egg sack was also included, which could explain the overprediction as egg concentrations were predicted to be lower than those in the ovarian matrix. The predicted egg concentrations for TBBPA performed well with most data within a 2-fold error but consistently underpredicted the measured data reported by Nyholm et al.^46^

#### 3.3.4 Brain

BPZ brain concentrations were used for fitting of the brain-to-blood partitioning and therefore performance cannot be assessed in an unbiased manner. A previous model^27^ predicted that the BPA brain concentration was higher than the whole-body concentration, which would be of high concern in terms of toxicity. However, applying our model with partitioning based on the experimental BPZ data yielded 1 order of magnitude lower BPA concentrations in the brain than in the whole body. Time-course measurements of brain concentrations of bisphenols in zebrafish have not been performed before this study; thus, data on bisphenols other than BPZ are still needed to confirm model estimates. The QSPR model used for estimating partitioning seems to be overpredicting brain partitioning in the case of BPZ; thus, the fitted partitioning was applied for all bisphenols instead. Note, however, that applying the same partition coefficients between bisphenols adds uncertainty, although the predicted coefficients do not differ much between each other in the case of organs. To the best of our knowledge, only Fang et al.^44^ have reported measured brain levels of BPA in zebrafish (see discussion above), and data from this study were in the range of modeled data (see Figure 3).

It has to be noted that we considered highly variable biological data from different organs, sampling times, and studies, which adds uncertainty to the PBTK modeling. Nevertheless, the majority of predicted concentrations were within a 2-fold error, which is considered adequate for the purpose of risk assessment.^85^ For more generic fish models used for organic pollutants, a 10-fold error has been considered acceptable.^27,28^ A recent PBTK study modeled BPA and its two major metabolites in stickleback, zebrafish, and trout.^86^ This model showed improved performance as compared to the previous generic model^27^ and showed performance comparable to our model. The predictions of BPA glucuronic acid metabolite are of similar accuracies (mostly 2–5-fold error) to the present study despite different methodologies being employed for model calibration.

Our model presents a compromise between generic and compound-specific PBTK models as it focuses on a narrow group of environmental pollutants. Using a QSPR model to predict tissue-specific partitioning allows for extrapolation but also reduces accuracy. As discussed, liver partitioning could be fitted instead of predicted for BPA and BPAF, while that was not necessary for BPZ or for gonad partitioning of all three compounds. If experimental data will be available in the future for additional bisphenols, some of these parameters can be fitted as done for BPA and BPAF to yield higher compound-specific accuracy.

### 3.4 Predicting Kinetics of Environmentally Relevant Bisphenols

The PBTK model was used to calculate BCF values for all 11 selected bisphenols to assess and compare their environmental risk (Table 1). The most bioaccumulating bisphenol is estimated to be Bimox M. However, Bimox M data are uncertain as the log *K*_ow_ (9.06) is outside the applicability domain (0–8) for which the *P*_BW_ model (eq 1) was developed. Except for this compound, none of the bisphenols showed a BCF above 2000, which would classify as bioaccumulating,^87^ indicating relatively low risk solely in terms of bioaccumulation potential. The predictions suggest that TBBPA, BPAP, BPC, and BPZ have higher bioaccumulation potential in the whole body as compared with BPAF, BPS, and BP-2. Overall, the BCF predictions follow trends observed in various other fish species, indicating that the model could be used for ranking bisphenols in terms of bioconcentration.^6,22,88^ Wang et al.^22^ studied the accumulation of bisphenols in carp exposed to a mixture, showing the highest internal concentrations for BPAF, BPAP, BPZ, and BPC as well as the lowest internal concentrations for BPS. BPB and BPA showed similar accumulation in carp but higher accumulation than BPS. Our predictions show good agreement with these findings, with the exception of BPAF. However, zebrafish studies have shown that BPAF has bioaccumulation potential similar to or lower than BPA (Table 2), which could either indicate interspecies variation for this compound or different toxicokinetics of BPAF when dosed in a mixture with other bisphenols. Additionally, a study in various lake fish species^6^ showed the highest bioaccumulation for BPC and BPZ at similar levels, followed by BPAF, then BPF and BPA showing comparable values, and lastly BPS, which is also in line with our model predictions except for BPAF. An investigation of bioaccumulation factors in marine fish revealed higher factors for BPAF and BPF than for BPA and BPS, which also agrees well with our predictions with the exception of BPA.^88^ The developed PBTK model was also used to estimate organ-specific BCFs, which indicated that the highest accumulation in the liver is expected for BPAF, BPA, and BPZ. Relatively low bioaccumulation was noted in the brain with the highest BCFs for TBBPA, BPAP, BPC, and BPZ. Ovaries generally showed higher accumulation than that in the whole body, with the compounds predicted to have the highest accumulation being TBBA, BPZ, and BPAP.

#### 3.4.1 Environmental Application

The model developed in this study was able to predict bioconcentration potentials of various bisphenols covering critical organs including the liver, gonads, and brain as well as the whole body. These organs are of high importance when it comes to potential endocrine disruption.

In fish, the liver is the main production site of the egg-yolk protein vitellogenin upon estrogenic activity.^3^ Vitellogenin is used as a biomarker for estrogenic and anti-estrogenic effects, and changes in serum levels have been related to various reproductive adversities in fish.^89^ Changes in vitellogenin levels have been observed in zebrafish upon exposure to BPA, BPAF, BPF, TBBPA, and BPS.^90,91^ Effects related to gonads have been observed upon exposure to BPA for both male and female zebrafish.^92,93^ Bisphenols that reach the ovaries distribute to eggs, causing exposure to offspring, which could result in effects on development as indicated by numerous studies.^24,94^ Similarly, our data suggest that only a small fraction of bisphenol distributes to the brain. However, various studies on zebrafish embryos on several bisphenols have shown alteration of normal brain function and development in zebrafish lasting to adult life-stages, making it a sensitive target organ of toxicity for bisphenols.^95−97^

The current PBTK model could be applied for various other compounds with similar structural and chemical characteristics as studied bisphenols. However, there are several important chemical parameters that are critical, including log *K*_ow_, which was used to calculate partition coefficients, fraction unbound in blood limiting elimination, and metabolic clearance rate as demonstrated by the global sensitivity analysis (Figures S6 and S7). In the present study, we employed fish-specific metabolic rates for parameterization of a fish PBTK model, reducing the uncertainty related to this parameter in particular to explain differences in kinetics between the different bisphenols. Furthermore, using predictive modeling for organ partitioning, it is possible to parameterize new models for these compounds on a variety of fish species based on their specific physiology. The ranking of bisphenols and similar compounds based on estimated BCFs can be then used in combination with toxicity data to identify emerging compounds of high concern, which display both high bioaccumulation and high toxicological activity. Data derived in the current study and the refined PBTK model thus provide a critical component in future environmental risk assessments of currently produced bisphenol-like compounds.

## Supporting Information Available

Additional methodological details and in-depth results (PDF)

## Notes

The model can be found on GitHub (https://github.com/ioanachelcea/PBTK_Adult_Zebrafish).
