# Supplementary material for: Physiologically Based Toxicokinetic Modeling of Bisphenols in Zebrafish (Danio rerio) Accounting for Variations in Metabolic Rates, Brain Distribution, and Liver Accumulation
Source: Environ Sci Technol. 2022 Jul 7;56(14):10216–28. doi: 10.1021/acs.est.2c01292 (PMC9301920; doi:10.1021/acs.est.2c01292)
Supplement: Supplementary file 1 — es2c01292_si_001.pdf [file es2c01292_si_001.pdf]

Physiologically Based Toxicokinetic Modelling of Bisphenols in Zebrafish (*Danio rerio*) Accounting for Variation in Metabolic Rates, Brain Distribution and Liver Accumulation

Ioana Chelcea[a], Stefan Örn[b], Timo Hamers[c], Jacco Koekkoek[c], Jessica Legradi[c], Carolina Vogs[b,d], Patrik L. Andersson[a]*

Author addresses

[a]Department of Chemistry, Umeå University, SE-901 87, Umeå, Sweden

[b]Department of Biomedical Sciences and Veterinary Public Health, Swedish University of Agricultural Sciences, Box 7028, SE-75007 Uppsala, Sweden

[c]Vrije Universiteit Amsterdam, Dept. Environment & Health; 1081 HV Amsterdam; The Netherlands

[d] Institute of Environmental Medicine, Karolinska Institutet, Stockholm, Sweden

Number of pages: 72

Number of figures: 7

Number of tables: 20

Contents

1. Bisphenol selection....3
2. Biotransformation rate estimation methodology....6
3. *In Vivo* Zebrafish Kinetics of BPZ....7
4. Analysis....8
4.1 LC-MS/MS method metabolism of bisphenols....8
4.2. LC-MS/MS method BPZ in zebrafish and water....11
4.3 Performance characteristics Bisphenol Z in zebrafish....14
5. Physiologically-based Toxicokinetic Modelling of Adult Zebrafish....15
5.1 Dynamic sub-models....16
5.2 Toxicokinetic modelling of metabolites....17
5.3. Parameters....19
5.4. Fitting methodology....24
6. Biotransformation rate estimation results....26
7. BPZ *in vivo* data....28
8. Predicting toxicokinetics of metabolites....30
10. PBTK predictions of previous models....31
11. Sensitivity analysis....31
12. R Script....32
13. References....67

1. Bisphenol selection

The bisphenol selection was based on a previous study by the Swedish Chemicals Agency (KEMI) [1] aiming to compile a list of bisphenols registered under REACH by 2017. The list included a total of 214 bisphenols out of which 39 were deemed to be relevant to consumers based on patent information[2] and thus likely to end up in environmental matrices.

In the selection process, bisphenols were defined as any substance with 2 phenol rings connected by a bridge at position 4 of each ring, where the bridge may constitute of one to nine carbon atoms or a single other atom, such as sulfur in the case of BPS and with the possibility of additional branches on this bridge. Furthermore, compounds with various functional groups and atoms bound to the phenyl rings were also included such as bromines in the case of tetrabromobisphenol A (TBBPA).

We expanded the list of 214 bisphenols published by KEMI[2] by performing a literature search in google scholar with the search terms: "(Bisphenols) AND (Analogues OR Analogs)" on 20th of January 2020. The first 50 articles of the search were screened for bisphenols not included in the KEMI list. Notable additions were BPB and BPC which are not registered for European use but are used in personal care products (PCPs) on Asian markets.[3]

Exclusion from the comprehensive list of 239 bisphenols was done according to the following criteria: pharmaceuticals for either human or veterinary use, chemicals banned from production, known carcinogens, intermediates, compounds with less than 100 references in SciFinder (unlikely to be produced in high volumes)[4] and various salts of already included bisphenols (e.g. sodium salt of BPS).

From the remaining 36 compounds a selection of 10 bisphenols was made for which relatively high human and exposure levels are expected (see further detail below), ER agonistic properties have been suggested, and in vivo toxicokinetic data in zebrafish have been reported (as described in further detail in the SI section 2). Lastly, Bimox M, a bisphenol known to be inactive on the ER but with high exposure risk was added to the list resulting in a total of 11 bisphenols (Figure S1 and Table 1).

Exposure risk was judged based on a ranking performed by KEMI, where bisphenols were considered of high exposure risk if patent information implied direct human exposure such as use in food-contact materials, receipt papers, toys or PCPs. Furthermore we deemed compounds to be environmentally-relevant if they have been identified in environmental compartments such as water and soil[5–9] or detected in PCPs outside the EU market.[3] Estrogenic activity was compiled based on experimental data if available from literature or high throughput screening

programs such as Tox21[10] or based on QSAR predictions.[2] A compound was considered as having estrogenic activity if it's EC_{50} for ER activation was below 1.5 μM with exception of TBBPA which was included due to availability of zebrafish *in vivo* data for validation purposes.

In total 10 ER-active bisphenols and one bisphenol that is known to be inactive on ER (Bimox M) were selected based on primarily risk for exposure to the environment and humans (Table S1). Measured physicochemical property data such as molecular weight (MW), log octanol-water partitioning (log K_{ow}), acid dissociation constant (pKa) and water solubility (W_{sol}) were collected from literature, if available. If measured data was unavailable, properties were predicted using *EPISUITE*[11] for W_{sol} and Jchem[12] for log K_{ow} and pKa. Additionally, measured or predicted plasma protein unbound fraction ($F_{unbound}$) in human or rats was also collected from the CompTox Dashboard. [13] Physicochemical properties for each selected bisphenol are shown in Table 1 and Table S10.

BPA, BPS, BPF, BPAF, BPAP, BPC, BPB and BPZ are all registered for polymer plastic synthesis, as coating and resins with BPB, BPC and BPS also being used in food packaging materials.[2] BP-2 is currently being used as a UV-blocker in cosmetics[14], while TBBPA is a flame-retardant added to various products such as carpets or furniture[15] and Bimox M is used in car lubrication.[2] All the selected bisphenols have been predicted or measured as ER active

with exception of Bimox M (Table S1). With exception of Bimox M, the selected compounds have been detected in various environmental matrixes or living organisms including humans and are therefore considered of environmental relevance.[16–19]

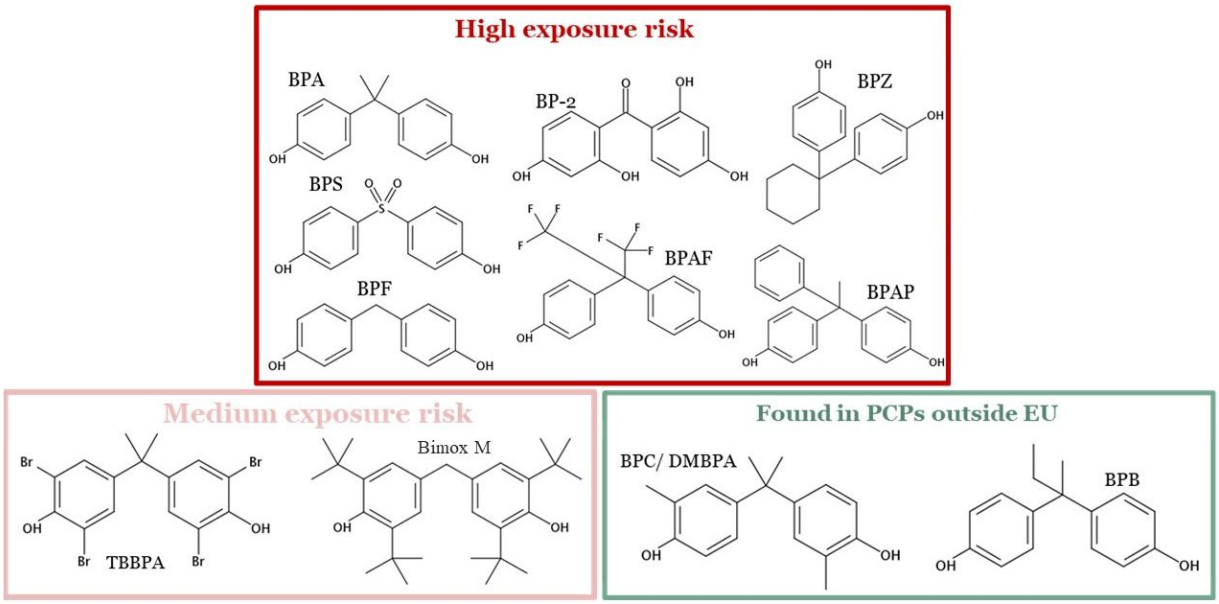

Figure S 1 Chemical structures of selected bisphenols

Table S 1 Selected bisphenols with information about their industrial use and estrogenic properties in terms of EC_{50} for Estrogen receptor (ER)

Name	CAS	Use[a]	BPA replacement	EC_{50} (μM)[b]	EC_{50} (μM)[c]	EC_{50} (μM)[d]
BPA	80-05-7	Polymers, thermal papers, food packaging		0.63	0.10	0.12
BPAP	1571-75-1	Polymer synthesis (insulation, coatings, resins etc.)	y		0.29	0.12
BPF	620-92-8	Polymers (insulation, coatings, resins etc.)	y	1.00	0.78	0.95
BPS	080-09-1	Polymers, (thermal) paper, food packaging	y	1.10	1.42	0.80
BPZ/BPCH	843-55-0	Polymers (resins, packaging etc)[e]		0.21		
BP-2	131-55-5	UV-blocker (Cosmetics, plastics, cars)			0.62	0.62
BPAF	1478-61-1	Polymers (insulation, coatings, resins etc.)		0.05	0.09	0.36
TBBPA	79-94-7	Flame-retardant (electronics, paper, plastics, carpets, furniture)		19.0	4.36	27.38
BPC/DMBPA	79-97-0	Polymers, thermal papers, food packaging		0.42	0.29	0.30
BPB	77-40-7	Polymers, food packaging		0.07	0.17	0.13
Bimox M	118-82-1	Lubricant (car engines)			1.00E+06	1.00E+06

[a]Based on information by the Swedish chemical agency[2]; [b]ERE-luciferase reporter assay using MCF-7 cells by Kitamura et al. 2005[20]; [c]ATG ERE CIS up assay from Tox21[10]; [d]ATG Era Trans up assay from Tox21[10]; [e]PubChem patent information[21]

2. Biotransformation rate estimation methodology

Rainbow trout liver S9 homogenate pooled from 3 male and 3 female donor fish; (1mg/mL Trinova Biochem; Lot: 180216) was pre-incubated at 11˚C in K_2HPO_4/KH_2PO_4 buffer (pH=7.8; 1 mL final volume) containing alamethicin from *Trichoderma viride* (25 μg/mL), and cofactors NADPH (2 mM), UDPGA (2 mM), GSH (5 mM), and PAPS (0.1 mM). After 10 min

109 of preincubation, biotransformation was started by adding 5 µL of bisphenol test compound
110 dissolved in methanol (1 µM final concentration). At t = 0, 2, 5, 15, 30, 60, 90, and 120 min, a
111 50 µL subsample was transferred from each duplicate incubation mixture to a centrifuge
112 microtube and mixed with 200 µL of methanol to denature the proteins and stop further
113 biotransformation.

114 First order disappearance rate constants did not differ significantly between replicate
115 experiments, except for BPA ($p=0.042$) and BPS ($p=0.00023$). For these two compounds,
116 relatively high levels of remaining parent compound were found in one replicate experiment at
117 t=90 min, where both observations were above the log-linear regression line. Despite the fact
118 that these observations could statistically not be qualified as outliers, they were removed from
119 the log-linear regression. After removal, replicate disappearance rate constants did no longer
120 differ for BPA ($p=0.32$), but did for BPS ($p=0.025$).

121

122 3. *In Vivo* Zebrafish Kinetics of BPZ

123 Adult zebrafish were purchased from a local supplier in Uppsala, Sweden, and acclimatized for
124 6 weeks before the start of the study. The zebrafish were kept in 26 ˚C charcoal filtered tap

125 water at a 12:12 h light/dark cycle. Fish were fed daily with commercial flake food (SERA
126 Vipan). A BPZ stock solution of 100 mg/L was created by dissolving crystal form BPZ in
127 methanol. The final solution was made by diluting the stock solution with charcoal-filtered tap
128 water to reach a final BPZ concentration of 1 mg/L. For the experiment, female fish were
129 selected and transferred into a 40 L tank with a flow-through system set at three daily water
130 volume renewals. Exposure to BPZ was done using an ISMATEC 24 peristaltic pump set at
131 6.3% capacity with a flow rate of 1.1 L/day and a stock solution of 1 mg BPZ/L. The BPZ stock
132 solution was kept in a 5-L glass flask stored in the dark.

133 For the depuration phase the fish were transferred into a new 40-L clean tank with identical
134 water flow-through regime without BPZ exposure. The concentration of BPZ was selected
135 based on previous kinetic studies on other bisphenols[22,23] which therefore would allow for easier
136 comparison of result. The use of animals and procedures described in this study were approved
137 by the regional ethical committee for animal experimentation (Dnr.5.8.18-008/2017).

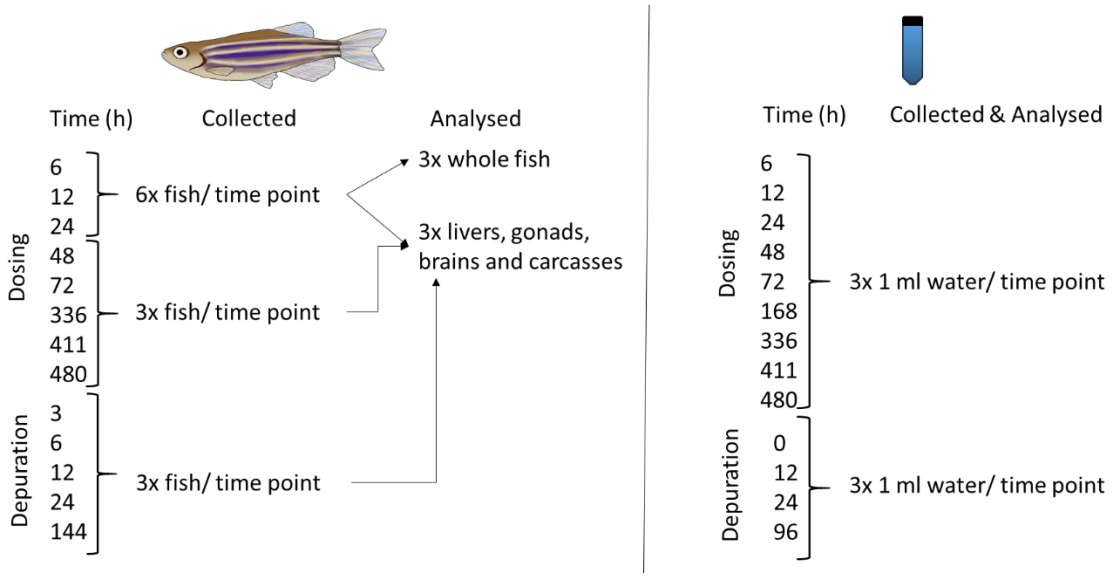

Figure S 2 Sampling and analysis schematic of BPZ for the in vivo zebrafish experiment

4. Analysis

4.1 LC-MS/MS method metabolism of bisphenols

From each microtube containing denaturated incubation mixture in methanol, a 50 µl aliquot was taken to which 10 µl ^{13}C or deuterium labeled internal standard was added, together with 150 µl 0.7% formic acid (for TBBPA, BPS, BP-2 and BPF) or 150 µl water (all other bisphenols). The samples were centrifuged at 9000g for 10 minutes and 10 µl supernatant was injected on a LC (Elute, Bruker) coupled with HESI source operating in the negative ion mode prior to triple quadrupole mass selective detector (EVOQ, Bruker). The bisphenols were

149 separated on an XBridge BEH C18 XP Column (2.5 µm, 2.1 x 100 mm; Waters, The

150 Netherlands) set at 40˚C, by applying a gradient of NH4F (0.2 mM) and methanol. LC

151 settings and MS/MS settings are given in Tables S2-S4.

152

153 Table S 2 LC system information for the metabolism experiments

System	Elute (Elute Bruker, Bremen Germany)		
Injection volume	10 µl		
Temperature sample tray	4˚C		
Temperature LC column	40˚C		
LC column	XBridge BEH C18 XP Column (2.5 µm, 2.1 x 100 mm) (Waters, Etten-Leur, The Netherlands)		
Gradient solvent		0.2 mM NH4F	Methanol
Time (min)	Flow (ml/min)	%	%
0	0.3	60	40
0.1	0.3	60	40
15	0.3	5	95
27	0.3	5	95
27.2	0.3	60	40
30	0.3	60	40

154

155 Table S 3 Source settings for metabolism experiments

System	EVOQ Elite (Bruker, Bremen Germany)
Source settings	
Spray voltage	-4500
Cone temperature	350
Cone gas flow	25
Probe temperature	350
Probe gas flow	40
Nebulizer gas flow	60
Probe and nebulizer gas	Nitrogen
Collision gas	Argon

156

157

158 Table S 4 MS/MS settings used for the biotransformation experiment

Compound	Precursor	Product ion	Collision energy	
BPA	227.0	212.0	12	Target
		133.0	21	Qualifier
BPA d16	241.0	233.0	12	
BPB	241.1	212.0	13	Target
BPB 13C12	253.1	221.8	13	

BPF	199.1	93.10	19	Target
		105.1	18	Qualifier
BPF 13C12	211.1	99.10	19	
BPAF	335.1	264.9	15	Target
		177.0	45	Qualifier
BPAF d4	339.1	268.8	15	
BPAP	289.0	274.0	17	Target
BPAP d5	294.0	279.0	17	
BP-2	240.0	135.1	13	Target
		109.1	17	Qualifier
BPZ	267.0	173.1	21	Target
		223.0	26	Qualifier
BPZ 13C12	279.0	179.1	21	
BPS	249.2	92.10	30	Target
		108.1	25	Qualifier
BPS 13C12	261.1	114.0	25	
BPC	255.2	240.3	18	Target
		147.3	24	Qualifier
BPC-2	279.0	35.40	11	Target
TBBPA	542.7	79.10	48	Target
		290.8	32	Qualifier

TBBPA 13C12	554.8	430.6	32	
Bimox M	423.2	407.2	43	

159

160 ## 4.2. LC-MS/MS method BPZ in zebrafish and water

161 Extract preparation

162 The fish organ tissues were transferred to a 1.5 ml Precellys vials with 1.4 mm ceramic beads
163 followed by the addition of internal standard and 1 ml acetonitrile. Tissue samples were then
164 homogenized using a Precellys 24 dual device operating for two cycles of 10 seconds at a speed
165 of 6500 rpm. The supernatant was then transferred into a new tube and this extraction process
166 was repeated. The whole zebrafish and carcass samples were manually cut and then sonicated
167 with internal standard and 5 ml acetonitrile for 30 minutes. The extraction was repeated with 5
168 ml acetonitrile. Volume of the combined extracts was reduced to 2 ml by a nitrogen gas stream
169 followed by dilution in 2 ml 5% NH_4OH.

170 BPZ in water samples was isolated by solid phase extraction using 150 mg Oasis MAX
171 cartridges. The cartridges were conditioned with 4 ml 2% formic acid in methanol, 4 ml
172 methanol and 4 ml water. After loading the samples, cartridges were washed with 4 ml 5%

173 NH_4OH and 4 ml acetonitrile/tetrahydofuran (1:1) mixture. The 4 ml 2% formic acid in

174 methanol eluates were evaporated until dry. Remaining residues were then reconstituted in 50

175 µl water and methanol 50/50 v/v mixture. Extracts were stored at 4˚C until measurement.

176 The extracts were measured on a LC (ExionLC, Sciex) coupled with a Turbo V Ion source (ESI)

177 operating in the negative ion mode prior to triple quadrupole mass selective detection (6500+,

178 Sciex). Separation of compound was done on a phenyl-hexyl column (Kinetex, 100 x 2.1 mm,

179 1.7 µm, Phenomenex) set at 45˚C using a gradient of 0.2 mM NH_4F and 0.2 mM NH_4F in

180 acetonitrile. The applied MRM transitions and MS/MS settings are described in Tables S5-S8.

181 Table S 5 LC system information for samples of the BPZ in vivo experiment

System		ExionLC, (Sciex)	
Injection volume		5 µl	
LC column		Kinetex Phenyl-hexyl column, 1.7 µm, 2,1 mm X 100 mm (Phenomenex)	
Delay column		Isolator column, 2.1 mm X 100 mm (Waters)	
Gradient solvent		0.2 mM NH4F	0.2 mM NH4F in Acetonitrile
Time	Flow (ml/min)	%	%
0	0.4	90	10
0.1	0.4	90	10
1	0.4	80	20
7	0.4	60	40

11	0.4	28	72
11.2	0.4	2	98
19	0.4	2	98
19.2	0.4	90	10
22	0.4	90	10

182

183 Table S 6 MS system information for samples of the BPZ in vivo experiment

System	6500+ (Sciex)
Source settings	
Ionspray voltage	- 4500 V
Temperature	500˚C
Curtain gas	35
Nebulizer gas	80
Heater gas	50

184

185 Table S 7 Transitions for information for samples of the BPZ in vivo experiment

	Precursor	Product ion	Declustering potential(V)	Collision energy(V)
BPZ	267	173	-165	-38
BPZ Qualifier	267	223	-165	-46
BPZ 13C	279	179	-165	-38

186

187

188

189 ## 4.3 Performance characteristics Bisphenol Z in zebrafish

190 Prior to the analysis of the zebrafish samples, the recovery and repeatability of the analytical
191 method was determined using fortified homogenized zebrafish tissue. These sample materials
192 were fortified to a level of 2 ng/g and were analyzed in eightfold. 22 blanks were analyzed
193 simultaneous with the samples. The data of blank measurements were used to determinate the

194 limit of detection (LOD). The LOD is defined as three times the standard deviation of the blank

195 plus the average blank value. The limit of quantification (LOQ) is defined as 3.3 * LOD value

196 All results of the validation and the quality control are presented in the following table.

197 Table S 8 Validation and quality control results for samples of the BPZ in vivo experiment

	Blank pg/extract	Recovery %	Repeatability %
	(n=22)	(n=8)	(n=8)
Bisphenol Z	5	78	4
Detection and report limits (calculated on based of average sample intake)			
	LOD ng/g	LOQ ng/g	
Whole fish	0.02	0.07	
Carcass	0.03	0.08	
Brain	2.4	7.8	
Liver	0.7	2.2	
Ovary	0.2	0.5	

198

199

200 5. Physiologically-based Toxicokinetic Modelling of Adult Zebrafish.

201 Table S 9 Experimental data used for PBTK model development and testing

Compound	Study	Species & Gender	Doses & Exposure route	Organs	Exposure	Exposure scenarios
BPZ	Current study	Zebrafish Females	17 µg/L in water	Liver, gonads, brain, carcass & whole body	20 days expsoure 4 days depuration	BW = 0.75 g T = 26 ˚C C_{water} = 0.017 µg/ml
BPA & BPA-GA	Lindholst et al. 2003[24]	Zebrafish Gender not specified	97.5 µg/L in water	Whole body	7 days exposure 7 days depuration	BW = 0.82 g (Females), 0.42 g (Males) T = 27 ˚C C_{water} = 0.0975 µg/ml
BPA & various PCPs	Chen et al. 2017[22]	Zebrafish Females	1.94 & 5.72 µg/L in water	Liver, gonads & whole body	6 days exposure 8 days depuration	BW = 1 T = 25 ˚C C_{water} = 0.00572 and 0.00194 µg/ml
BPA	Fang et al. 2016[25]	Zebrafish Males and females	2-0.7, 20-2.6 & 200-51 µg/L*	Liver, gonads, brain	21 day exposure	BW = 0.82 g (Females), 0.42 g (Males) T = 28 ˚CC_{water} = 0.002, 0.02, 0.2 µg/ml*
BPAF & BPAF-GA	Shi et al. 2016[26]	Zebrafish Males and females	20 µg/L in water	Liver, gonads & whole body	7 days exposure 3 days depuration	BW = 0.82 g (Females) and 0.42 g (Males) T = 27 ˚C C_{water} = 0.02 µg/ml

						BW = 1.40 g<br>T = 26 ˚C
TBBPA	Nyholm et al. 2009[27]	Zebrafish<br>Females	10 and 100 nmol/g dry weight in feed	Eggs & whole body	43 days expsoure	Amount fed twice daily = (5.438*(0.02)*BW)/2 and (54.38*(0.02)*BW)/2

For BPAF, the provided BCF values were calculated to internal concentrations (µg/g bodyweight) using the nominal water concentration. For TBBPA, the amount in food was calulated based on the information that the daily amount of food (g) was 2% of the body weight and the fish were fed twice daily (half a dose every 12 h). The internal concentrations for this study were transformed from nmol/g lipid weight to µg/g body weight based on the lipid content in female fish (10% of wet weight)[28] *or the lipid content in eggs (0.47 % of wet weight) measured by Nyholm et al.*[27]*. For Lindholst et al. predictions were done for both the female and male models and an average of the concentration predictions was used to assess model performance. Note all studies covered several time-points in order to reach steady state.*Doses in Fang et al. study varied by up to 87% before and after daily water renewals thus the modelled volume of the water was adjusted and dose was renewed every 24h in order to capture the observed variation to some extent. For modelling it was assumed that this variation was consistent throughout the whole 21 days exposure.*

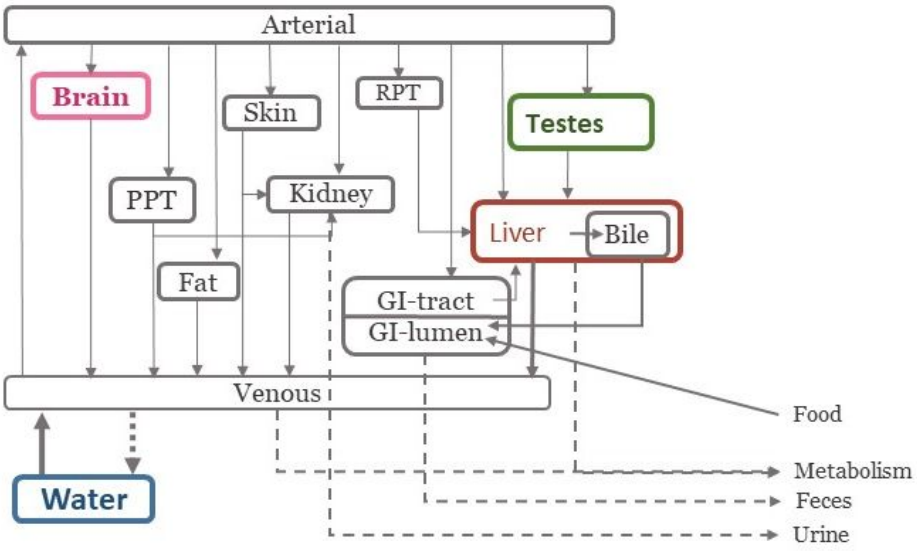

Figure S 3 PBTK model structure for adult male zebrafish adopted from Grech et al.[29] Solid arrows represent mass balance flows between organs and dashed arrows represent possible elimination pathways. Colored compartments represent organs for which experimental data were available. Abbreviated compartment names are richly perfused tissue (RPT), poorly perfused tissue (PPT) and gastro-intestinal (GI)–tract/-lumen.

5.1 Dynamic sub-models

The temperature model enables adjustments for T-dependent changes of cardiac output (F_{card}) and oxygen consumption rate (VO_2) as described by Barron et al. [30] and by Wood and Shelton.[31]

$$\dot{k}_T = \dot{k}_r * \dot{A}_T \text{ with } \dot{A}_T = \exp\left(\frac{T_A}{T_r} - \frac{T_A}{T}\right) \#(S1)$$

Where k_r is the value of the reaction rate, k_T at the temperature T_r. T_r is the temperature at which the reference value was measured while T is the absolute temperature and T_A represent the Arrhenius temperature in Kelvin.

The growth model is based on dynamic energy budget theory (DEB) and accounts for the increase in mass over the duration of the simulation as described by Kooijman et al.[32] and Grech et.al.[29]

$$V = L^3 = (\delta * L_w)^3 \quad (S2)$$

$$\frac{dL}{dt} = \frac{\dot{v}}{3 * (f + g)} * \left(M * f - \frac{L}{L_m}\right) \#(S3)$$

where V is the volume (ml), L the structural length (mm), δ the shape coefficient and L_w the physical length (mm). The change in length over time is then described by equation S2, where v is the energy conductance (mm/d) which is dependent on temperature as described in equation S3, f is the relative food density, g the energy conductance ratio, Lm the maximum structural length and M is the ratio of cumulate energy invested in maturity at metamorphosis (J) over the cumulate energy invested in maturity at birth (J). In the case of zebrafish M was set 1 as to describe a Bertalanffy growth curve.

5.2 Toxicokinetic modelling of metabolites

In the case of BPA and BPAF, experimental data were available for the main metabolites, BPA glucuronic acid (BPA-GA)[24] and BPAF glucuronic acid (BPAF-GA)[26] (Table S9). The same PBTK structure as for the other bisphenols was used to also simulate concentration of these

metabolites. Metabolite input occurred via liver at the same rate as the clearance of parent compound and elimination was modeled to occur via bile. Furthermore, tissue partitioning for metabolites was parameterized using models and Bertelsen et al.[33] based on log K_{ow} values predicted with EpiSuite . The liver-bile excretion (Ke_{bile}) and bile-gut (K_{BG}) excretion rates were fitted to data from Lindholst et al.[24] in the case of BPA-GA. These rates for BPAF-GA were fitted on the male data from Shi et al.[26] allowing us to use the female data set for validation. Both fittings were done using the Nelder-Mead algorithm with the aim to minimize the squared sum of residuals. The liver-bile excretion rate was set in the ranges of 0 to 1000 while the bile-gut excretion was set in the range of 0 to 10^{12} and the initial estimates were varied at 0, 1, 10, 100 and 1000 for Ke_{bile} and at 0, 10, 100, 1000 and 10^{12} for K_{BG}. Kebile and KBG were chosen as the major route of elimination for glucuronides as these type of metabolites have been suggested to be eliminated primarly via bile in fish.[24,34,35] Metabolite kinetics of the remaining bisphenol metabolites was not simulated due to lack of experimental data to compare with.

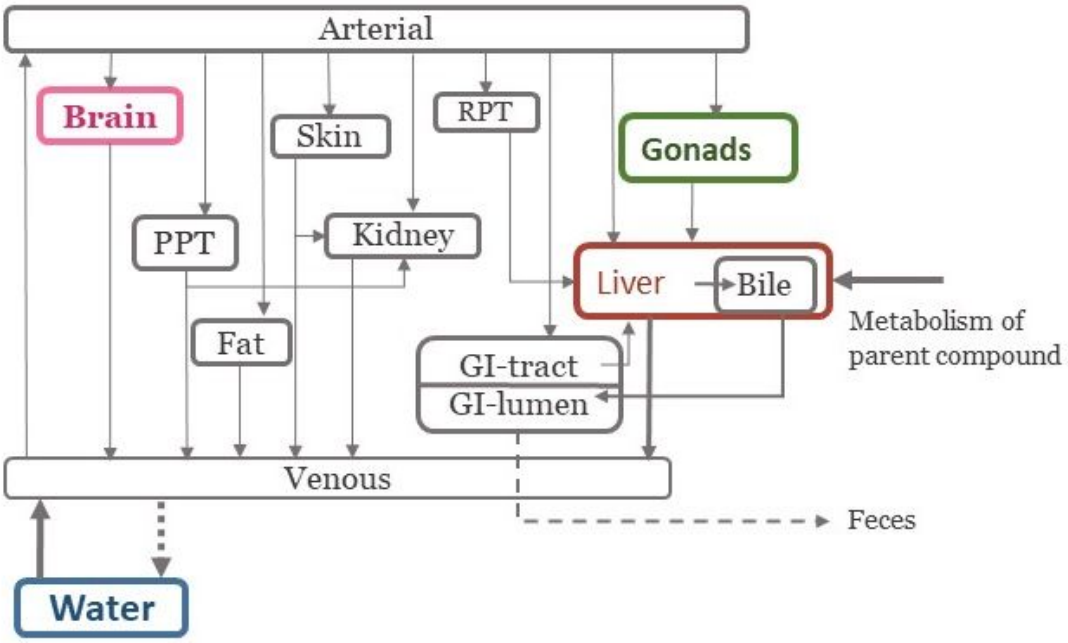

255

256 Figure S 4 PBTK model structure for BPA-GA in adult zebrafish adopted from Grech et al.[29] Solid arrows represent

257 mass balance flows between organs and dashed arrows represent possible elimination pathways. Colored

258 compartments represent organs for which experimental data were available. Abbreviated compartment names are

259 richly perfused tissue (RPT), poorly perfused tissue (PPT) and gastro-intestinal (GI)–tract/-lumen.

260 5.3. Parameters.

261 Table S 10 Male physiological parameters used for PBTK model

Parameter	Description	Reference	Mean
BW_i	Initial body weight	Grech et al. 2018	0.41
liv_frac	Blood flow fraction to liver	Grech et al. 2018	0.01
gon_frac	Blood flow fraction to gonads	Grech et al. 2018	0.07
git_frac	Blood flow fraction to GI-tract	Grech et al. 2018	0.17
fat_frac	Blood flow fraction to fat	Grech et al. 2018	0.01
brain_frac	Blood flow fraction to brain	Grech et al. 2018	0.06

kidney_frac	Blood flow fraction to Kidney	Grech et al. 2018	0.02
skin_frac	Blood flow fraction to skin	Grech et al. 2018	0.06
rp_frac	Blood flow fraction to richly perfused tissue	Grech et al. 2018	0.19
pp_frac	Blood flow fraction to poorly perfused tissue	Grech et al. 2018	0.41
art_ven_frac	Arterial to venous fraction	Grech et al. 2018	0.33
a_Fs	Blood flow fraction from skin to ven directly	Grech et al. 2018	0.10
a_Fpp	Blood flow fraction from ppt to ven directly	Grech et al. 2018	0.40
sc_blood	V scaling (fraction of BW)	Grech et al. 2018	0.02
sc_liv	V scaling (fraction of BW)	Current study	0.01
sc_gon	V scaling (fraction of BW)	Grech et al. 2018	0.01
sc_fat	V scaling (fraction of BW)	Grech et al. 2018	0.02
sc_git	V scaling (fraction of BW)	Grech et al. 2018	0.10
sc_brain	V scaling (fraction of BW)	Current study	0.01
sc_kidney	V scaling (fraction of BW)	Grech et al. 2018	0.002
sc_skin	V scaling (fraction of BW)	Grech et al. 2018	0.10
sc_rp	V scaling (fraction of BW)	Grech et al. 2018	0.03
sc_pp	V scaling (fraction of BW)	Grech et al. 2018	0.70
urine_rate	V_burst = 1.2 mL.kg-1 every 29.82 minutes proposed by Curtis 1991	Grech et al. 2018	0.06
urination_interval		Grech et al. 2018	0.02
a_BW_L	a relation BW(g) = F(L(cm))	Grech et al. 2018	0.009
b_BW_L	b relation BW(g) = F(L(cm))	Grech et al. 2018	3.24
OEE	O2 extraction efficiency	Grech et al. 2018	0.71
water_liver	Water content in liver	Grech et al. 2018	0.65
water_brain	Water content in brain	Grech et al. 2018	0.75
water_gonads	Water content in gonads	Grech et al. 2018	0.52
water_fat	Water content in fat	Grech et al. 2018	0.03
water_skin	Water content in skin	Grech et al. 2018	0.76
water_GIT	Water content in GIT	Grech et al. 2018	0.62
water_kidney	Water content in Kidney	Grech et al. 2018	0.49
water_rp	Water content in rpt	Grech et al. 2018	0.53
water_pp	Water content in ppt	Grech et al. 2018	0.69
lipids_liver	Lipid content in liver	Grech et al. 2018	0.11
lipids_brain	Lipid content in brain	Grech et al. 2018	0.07
lipids_gonads	Lipid content in gonad	Grech et al. 2018	0.22
lipids_fat	Lipid content in fat	Grech et al. 2018	1.00
lipids_skin	Lipid content in skin	Grech et al. 2018	0.05
lipids_GIT	Lipid content in git	Grech et al. 2018	0.04

lipids_kidney	Lipid content in kidney	Grech et al. 2018	0.17
lipids_rp	Lipid content in rp	Grech et al. 2018	0.07
lipids_pp	Lipid content in pp	Grech et al. 2018	0.06
Ke_bile	Bile excretion from liver to bile (1/d)	Not parameterized due to lack of data	0.00
K_BG	Rate from bile to GI (0-1E12) (1/d)	Not parameterized due to lack of data	1E+12
Ku	Oral absorption	Not parameterized due to lack of data (Except for TBBPA)	0.00
Ke_urine	Urine excretion (1/d)	Not parameterized due to lack of data	0.00
Ke_feces	Feces excretion (1/d)	Grech et al. 2018	0.83
Cl_plasma	Clearance in plasma (ml/d(ml)	Not parameterized due to lack of data	0.00
frac_absorbed	Fraction of food absorbed to GIT	Assumed to be 100% due to lack of data	1.00

262

263 Table S 11 Female physiological parameters used for PBTK model

Parameter	Description	Reference	Mean
BW_i	Initial body weight	Grech et al. 2018	0.82
liv_frac	Blood flow fraction to liver	Grech et al. 2018	0.02
gon_frac	Blood flow fraction to gonads	Grech et al. 2018	0.01
git_frac	Blood flow fraction to GI-tract	Grech et al. 2018	0.17
fat_frac	Blood flow fraction to fat	Grech et al. 2018	0.01
brain_frac	Blood flow fraction to brain	Grech et al. 2018	0.03
kidney_frac	Blood flow fraction to Kidney	Grech et al. 2018	0.02
skin_frac	Blood flow fraction to skin	Grech et al. 2018	0.06
rp_frac	Blood flow fraction to richly perfused tissue	Grech et al. 2018	0.13

pp_frac	Blood flow fraction to poorly perfused tissue (calculated)	Grech et al. 2018	0.55
art_ven_frac	Arterial to venous fraction	Grech et al. 2018	0.33
a_Fs	Blood flow fraction from skin to ven directly	Grech et al. 2018	0.10
a_Fpp	Blood flow fraction from ppt to ven directly	Grech et al. 2018	0.40
sc_blood	V scaling (fraction of BW)	Grech et al. 2018	0.02
sc_liv	V scaling (fraction of BW)	Current study	0.03
sc_gon	V scaling (fraction of BW)	Grech et al. 2018	0.08
sc_fat	V scaling (fraction of BW)	Grech et al. 2018	0.02
sc_git	V scaling (fraction of BW)	Grech et al. 2018	0.10
sc_brain	V scaling (fraction of BW)	Current study	0.01
sc_kidney	V scaling (fraction of BW)	Grech et al. 2018	0.00
sc_skin	V scaling (fraction of BW)	Grech et al. 2018	0.10
sc_rp	V scaling (fraction of BW)	Grech et al. 2018	0.03
sc_pp	V scaling (fraction of BW) (calculated)	Grech et al. 2018	0.61
spawnrate	How often female lays eggs (days)	Spence et al. 2008	1.50
Egggrowth	How quickly clutch grows (ml/d)	Estimated based on spawnrate and clutchsize (Castranova et al. 2011)	0.027
V_egg	Volume of one egg	Estimated based on egg diameter (Cartner et al. 2019)	2.12E-04
urine_rate	V_burst = 1.2 mL.kg-1 every 29.82 minutes proposed by Curtis 1991	Grech et al. 2018	0.06
urination_interval		Not parameterized due to lack of data	0.00
a_BW_L	a relation BW(g) = F(L(cm))	Grech et al. 2018	0.01
b_BW_L	b relation BW(g) = F(L(cm))	Grech et al. 2018	3.24
OEE	O2 extraction efficiency	Grech et al. 2018	0.71
water_liver	Water content in liver	Grech et al. 2018	0.69
water_brain	Water content in brain	Grech et al. 2018	0.76
water_gonads	Water content in gonads	Grech et al. 2018	0.65
water_fat	Water content in fat	Grech et al. 2018	0.03
water_skin	Water content in skin	Grech et al. 2018	0.76
water_GIT	Water content in GIT	Grech et al. 2018	0.62
water_kidney	Water content in Kidney	Grech et al. 2018	0.49

water_rp	Water content in rpt	Grech et al. 2018	0.53
water_pp	Water content in ppt	Grech et al. 2018	0.69
lipids_liver	Lipid content in liver	average from Grech et al. 2018 and Pery et. al 2013	0.07
lipids_brain	Lipid content in brain	average from Grech et al. 2018 and Pery et. al 2013	0.09
lipids_gonads	Lipid content in gonad	Grech et al. 2018	0.08
lipids_fat	Lipid content in fat	Grech et al. 2018	1.00
lipids_skin	Lipid content in skin	Grech et al. 2018	0.05
lipids_GIT	Lipid content in git	Grech et al. 2018	0.04
lipids_kidney	Lipid content in kidney	Grech et al. 2018	0.17
lipids_rp	Lipid content in rp	Grech et al. 2018	0.07
lipids_pp	Lipid content in pp	Grech et al. 2018	0.06
Ke_bile		Grech et al. 2018	0.00
K_BG		Grech et al. 2018	1.00E+12
Ku	Oral absorption	Not parameterized due to lack of data (except for TBBPA)	0.00
Ke_urine	Urine excretion (1/d)	Not parameterized due to lack of data	0.00
Ke_feces	Feces excretion (1/d)	Grech et al. 2018	0.83
Cl_plasma	Clearance in plasma (ml/d/ml)	Not parameterized due to lack of data	0.00
frac_absorbed	Fraction of food absorbed to GIT	Assumed to be 100% due to lack of data	1.00

264

265 Table S 12 *Parameters required for modelling maternal transfer.*[27,36–39]

	Mean	Reference
Egg diameter (mm)	0.74	Cartner et al. 2019
Egg V (ml)[a]	$2.12*10^{-4}$	
Egg lipid content (% of wet weight)	0.47	Nyholm et al 2007
Clutchsize (nr of eggs)	188	Mean of Castranova et al. 2011, Örn et al. 1998 and Spence et al. 2007
Clutch V (ml)	0.04	Based on Clutchsize and egg V

Spawnrate (days^{-1})	1.5	Mean of Spence et al. 2007 and Örn et al. 1998
Nr of eggs/day	125	
Clutch growth rate (ml/day)[b]	0.027	

[a] *Assuming egg is a perfect sphere and a density = 1;* [b] *Assuming linear growth*

Table S 13 Chemical parameters used for PBTK model

**Name**	**MW (g/mol)**	**WS[a] (mg/L)**	**Ratio blood-plasma[b]**	**$F_{unbound}$[c]**
BPA	229	$1.73*10^2$	1.08	0.035[d]
BPAF	336	4.30	0.86	0.010
BPAP	290	3.76	1.00[e]	0.020[f]
BPB	242	29.2	1.04	0.018
BPC	256	7.46	1.00[e]	0.020[f]
BPF	200	$5.43*10^2$	1.41	0.080[f]
BPS	250	$3.52*10^3$	1.48	0.058
BPZ	268	3.78	1.00[e]	0.020[f]
BP-2	246	$3.99*10^2$	1.00[e]	0.035
TBBPA	544	$1.00*10^{-3}$	1.00[e]	0.001
Bimox M	425	$1.71*10^{-4}$	1.00[e]	0.010[f]
BPA-GA	408	7.93*102	1.00[g]	1.000[g]
BPAF-GA	512	32.10	1.00[g]	1.000[g]

[a]Water solubility (WS) predicted by EpiSuite Wskowwin v 1.42; [b]Measured values by Grumetto et al 2019[40];

[c]Plasma fraction unbound (measured human values from CompTox Dashboard);[d]Measured by

Edginton&Ritter[41]; [f]Predicted value from CompTox Dashboard; [g] Measured by Staples et al. 1998[42];

Table S 14 Modelling parameters used to simulate BPA-GA and BPAF-GA

Name	Description	BPA-GA	BPAF-GA
M_log_Kow[a]	Octanol-water partitioning of main metabolit (Glucuronic acid conjugates)	2.1	2.83
M_Ke_bile	Bile excretion rate (1/d)	12.7[b]	61.7[c]
M_K_BG	Bile to gut excretion rate (1/d)	0.67[b]	1.49[c]

[a]*Predicted using EpiSuite Wskowwin v 1.42;* [b]*Fitted based on BPA-GA data by Lindholst et al.*[24]*;* [c]*Fitted based on BPAF-GA male data from Shi et al.*[26]

5.4. Fitting methodology

For fitting, the model was run using the exposure scenarios of each fitting data set. All parameters were fixed as presented in Tables S10-S14 with exception of the parameter for which fitting was performed. The outcome evaluated by fitting algorithm was the concentration over time in the same tissue as the experimental data. Outcomes used for fitting to measured experimental data were: concentration in brain for brain-blood partition coefficient, concentration in liver for liver-blood partition coefficient, concentration in eggs for egg-gonad partition coefficient and whole-body metabolite concentration for Ke_{bile} and K_{BG} of metabolites. For the liver, brain and egg partitioning, the range was set between 0.01 and 500 and the Nelder-Mead algorithm was run with initial estimates at 1, 5, 10, 50 and 500 in order to assure a global minimum was found. The fitted values returning the lowest squared sum of residuals were used as parameters. For BPA, P_{livb} was fitted on the liver concentrations measured in the low dose

(1.94 μg/L) exposure group from Chen et al.[22] In the case of BPAF, P_{livb} was fitted on the liver concentrations measured in the males.[26] The P_{livb} of the remaining bisphenols including BPZ were predicted using the Bertelsen et al.[33] model. P_{egg} was fitted based on the TBBPA egg concentration data from Nyholm et al.[27] from the high dose exposure group (100 nmol/g feed) and was then used to parameterize all bisphenols. The GI lumen to GI tract diffusion rate (K_u) was also fitted simultaneously using this same data but was only applied on TBBPA in order to validate on the low exposure group (10 nmol/g) from Nyholm et al. All fitted parameters, data used for fitting and the use of the fitted values are presented in Table S15.

Table S 15 Parameters that were fitted using the Nelder-Mead algorithm

Parameter	Description	Value	Used for predictions of	Range tested	Data used for fitting
P_{bb}	Brain to blood partition coefficient	0.42	All bisphenols	0.01-500	Brain BPZ concentrations in current study
P_{livb}	Liver to blood partition coefficient	195	BPA	0.01-500	Liver BPA concentrations in fish exposed to 1.94 μg/L BPA (Chen et al. 2017)
		70.0	BPAF	0.01-500	Liver BPAF concentrations in males exposed to 20 μg/L BPAF (Shi et al. 2016)
P_{egg}		1.58	All bisphenols	0.01-500	Egg TBBPA concentrations in fish exposed to 100 nmol/g feed TBBPA (Nyholm et al. 2009)
Ku	Diffusion coefficient from GI lumen into GI tract	8.40	TBBPA	0-1000	Egg TBBPA concentrations in fish exposed to 100 nmol/g feed TBBPA (Nyholm et al. 2009)
Ke_{bile}	Liver to bile excretion rate (1/d)	12.7	BPA-GA	0-1000	Whole body BPA-GA concentrations in fish exposed to 97.5 μg/L BPA (Lindholst et al. 2013)

K_{BG}	Bile to gut excretion rate (1/d)	61.6	BPAF-GA	0-1000	Whole body BPAF-GA concentrations in males exposed to 20 µg/L BPAF (Shi et al. 2016)
		0.70	BPA-GA	0-1E12	Whole body BPA-GA concentrations in fish exposed to 97.5 µg/L BPA (Lindholst et al. 2013)
		1.49	BPAF-GA	0-1E12	Whole body BPAF-GA concentrations in males exposed to 20 µg/L BPAF (Shi et al. 2016)

6. Biotransformation rate estimation results

Table S 16 Clearance (CL) rates of bisphenols measured in rainbow trout liver S9 fractions.

Each experiment was performed twice i.e. Series 1 (s1) and series 2 (s2)

BP	Comment	slope	intercept	k (1/h)	mg/mL protein	CL (mL/(h x mg))	slope	intercept	k (1/h)	mg/mL protein	CL (mL/(h x mg))	CL (mL/(h x mg))
BPA	removal of 90-min points in s1 due to outliying	-0.26	1.14	0.61	1.01	0.60	-0.29	1.04	0.66	1.01	0.65	0.63
BPB	120-min points were below LLOD	-0.41	0.99	0.95	1.04	0.92	-0.43	1.29	0.99	0.99	1.00	0.96
BPC		-0.45	1.25	1.03	1.01	1.01	-0.45	0.72	1.05	0.99	1.05	1.03
BPF		-0.14	0.86	0.31	1.01	0.31	-0.13	1.11	0.31	1.00	0.30	0.31
BPS	removal of 90-min points in s1 due to outliying	-0.18	0.94	0.41	1.03	0.40	-0.21	0.86	0.48	0.95	0.50	0.45
BPZ		-0.14	0.94	0.32	0.97	0.33	-0.11	1.13	0.26	0.98	0.26	0.30
BPAF		-0.32	1.15	0.73	0.98	0.75	-0.34	1.66	0.78	0.98	0.80	0.78
BPAP	>60-min points were below LLOD	-0.75	0.83	1.73	0.97	1.79	-0.76	1.06	1.74	0.95	1.82	1.80
BP2	>6-min points were below LLOD	-5.51	0.81	12.68	0.93	13.69	-6.32	0.93	14.56	0.91	15.92	14.80
BimoxM	no biotransformation	0.01	0.53	0.03	0.96	0.03	-0.02	0.99	0.04	1.02	0.04	0.03
TBBPA	biotransformation stops after 15 min. Only considered first 15 min	-1.72	1.29	3.95	1.01	3.90	-1.90	1.16	4.38	0.98	4.45	4.18

304

Table S 17 Clearance of Bisphenols in human hepatocytes, rainbow trout S9 fraction and corresponding *in vivo* intrinsic clearance

Compound	CL^a (ml/d/g liver)	CL^b (ml/d/g liver)	$CL_{in\ vivo,int}^c$ (L/d/Kg fish)
BPA	$2.45*10^3$	$1.46*10^5$	36.7
BPS	$1.77*10^3$	$2.43*10^5$	26.5
BPF	$1.20*10^3$	$1.87*10^5$	18.0
BPAF	$3.04*10^3$	$9.18*10^5$	45.5
BPZ	$1.16*10^3$	$1.93*10^{4\ d}$	17.4
BP-2	$5.79*10^4$	$1.41*10^5$	869
TBBPA	$1.63*10^4$	$3.71*10^5$	245
BPAP	$7.06*10^3$	$2.43*10^{5\ d}$	106
Bimox M	0	$2.00*10^{6\ d}$	0
BPC	$4.04*10^3$	-	60.6
BPB	$3.76*10^3$	$2.62*10^5$	56.4

[a]*in vitro* rainbow trout clearance rates measured in this study; [b]Human clearance rates from CompTox Dashboard database[13]. Calculated using the factor of 510 x 10^6 hepatocytes/g liver[43,44]; [c]In vivo intrinsic clearance rate using measured clearance from current study and transformed as suggested by Nichols et al.[45]; [d]Predicted values

318

319 7. BPZ *in vivo* data

320 Table S 18 Measured BPZ internal concentration in female zebrafish

Time (h)	Liver	Brain	Ovary	Carcass	Whole body (measured)	Whole body (calculated)
**Mean concentration (ng/g)**						
Control	<1.04	<3.9	<0.22	<0.02	<0.03	
6	181.7	72.0	85.3	159.7	214.7	129.5
12	57.5	82.1	112.7	261.2	336.1	203.3
24	363.5	115.8	145.5	670.7	435.3	529.5
48	225.2	64.4	146.3	612.0	NM	500.2
72	536.0	170.0	259.0	1120.7	NM	899.6
336	146.0	155.5	268.7	347.9	NM	295.8
411	231.8	150.2	214.2	557.0	NM	471.6
480*	1006.7	61.4	69.3	249.4	NM	449.2
483	251.7	14.4	108.4	133.1	NM	128.1
486	71.3	7.9	107.8	24.3	NM	35.1
492	11.0	<2.5	84.5	18.9	NM	28.2
504	12.4	<3	48.3	8.3	NM	14.5
576	4.4	<2.5	1.0	1.6	NM	1.5
**Standard deviation**						
6	23.2	7.8	14.7	42.7	56.8	28.0
12	24.9	38.6	12.6	113.5	56.3	81.0
24	395.3	14.9	10.9	277.1	178.3	172.0
48	144.6	9.7	30.1	215.9		169.7
72	530.3	57.6	51.8	155.5		86.8
336	17.4	37.8	37.5	53.0		33.3
411	73.6	25.8	52.6	92.6		90.8
480	890.3	8.9	61.6	105.4		340.9
483	242.6	4.1	34.3	82.7		71.8
486	40.9	2.3	28.9	8.2		6.2
492	1.6		6.8	1.7		4.1
504	1.0		22.6	4.5		6.3

576	1.6		0.2	1.1		0.9
			Coefficient of Variance			
6	0.1	0.1	0.2	0.3	0.3	0.2
12	0.4	0.5	0.1	0.4	0.2	0.4
24	1.1	0.1	0.1	0.4	0.4	0.3
48	0.6	0.2	0.2	0.4		0.3
72	1.0	0.3	0.2	0.1		0.1
336	0.1	0.2	0.1	0.2		0.1
411	0.3	0.2	0.2	0.2		0.2
480	0.9	0.1	0.9	0.4		0.8
483	1.0	0.3	0.3	0.6		0.6
486	0.6	0.3	0.3	0.3		0.2
492	0.1		0.1	0.1		0.1
504	0.1		0.5	0.5		0.4
576	0.4		0.2	0.7		0.6

**One fish was removed at this time-point as it was a statistical outlier for all four measured organs;*

NM= Not measured.

Table S 19 Water concentration for BPZ experiment

Time (h)	Mean water concentration (µg/L)	Standard deviation
0	10.1	1.5
12	15.7	1.2
24	12.3	0.5
48	16.3	1.2
72	14.0	0.8
168	16.7	0.9
336	20.7	1.2
411	23.0	0.8
480	24.3	1.2

**Total**	17.0	4.7

8. Predicting toxicokinetics of metabolites

Glucuronic acid conjugates for both BPA and BPAF showed levels far exceeding those of the parent compound *in vivo*.[24,26] Although the glucuronic acid conjugates of bisphenols have not shown any ER activity[46,47], their high internal concentrations combined with the possibility for de-conjugation[48,49] makes them relevant modelling targets. C_{max} and AUC of BPA-GA and BPAF-GA were accurately predicted by the model within a 2-fold error (Table S18) and in the case of BPAF-GA, the model over-predicted the male observations but under-predicted the females, thereby supporting the hypothesis of gender-specific clearance capacities.

Table S 20 Observed and predicted toxicokinetic results for BPA-GA and BPAF-GA

Study	Gender	Organ	C_{max}	$t_{1/2}$	AUC	
**BPA-GA**						
Lindholst et al. 2003	Female	Whole body*	13.3	3.11	106	Predicted
			16.2	8.16	109	Observed
**BPAF-GA**						
Shi et al. 2016	Male	Whole body*	0.78	-	4.07	Predicted
			0.60	-	3.54	Observed
	Female	Whole body	0.56	-	3.01	Predicted
			1.09	-	6.54	Observed

**Data used for fitting*

340

10. PBTK predictions of previous models

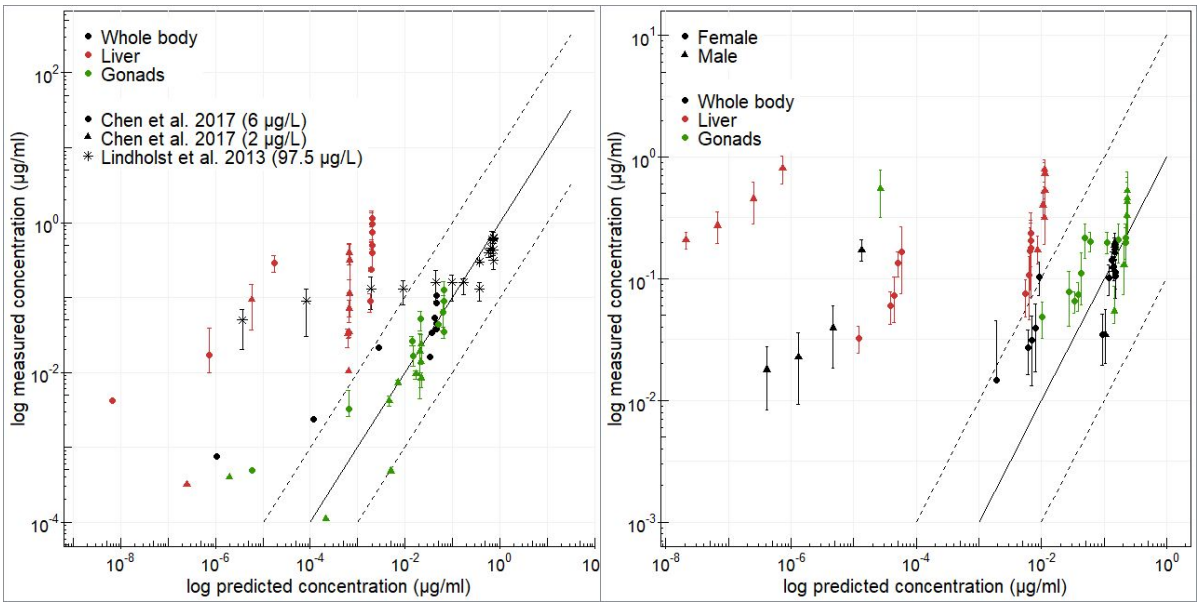

Figure S 5 Predictions of unmodified Grech et al.[29] model for BPA (left) data by Chen et al.[22] and Lindholst et al.[24] and for BPAF data by Shi et al. (using the same parameters as for BPA). Line represents perfect prediction and dotted lines represent 10-fold error.

11. Sensitivity analysis.

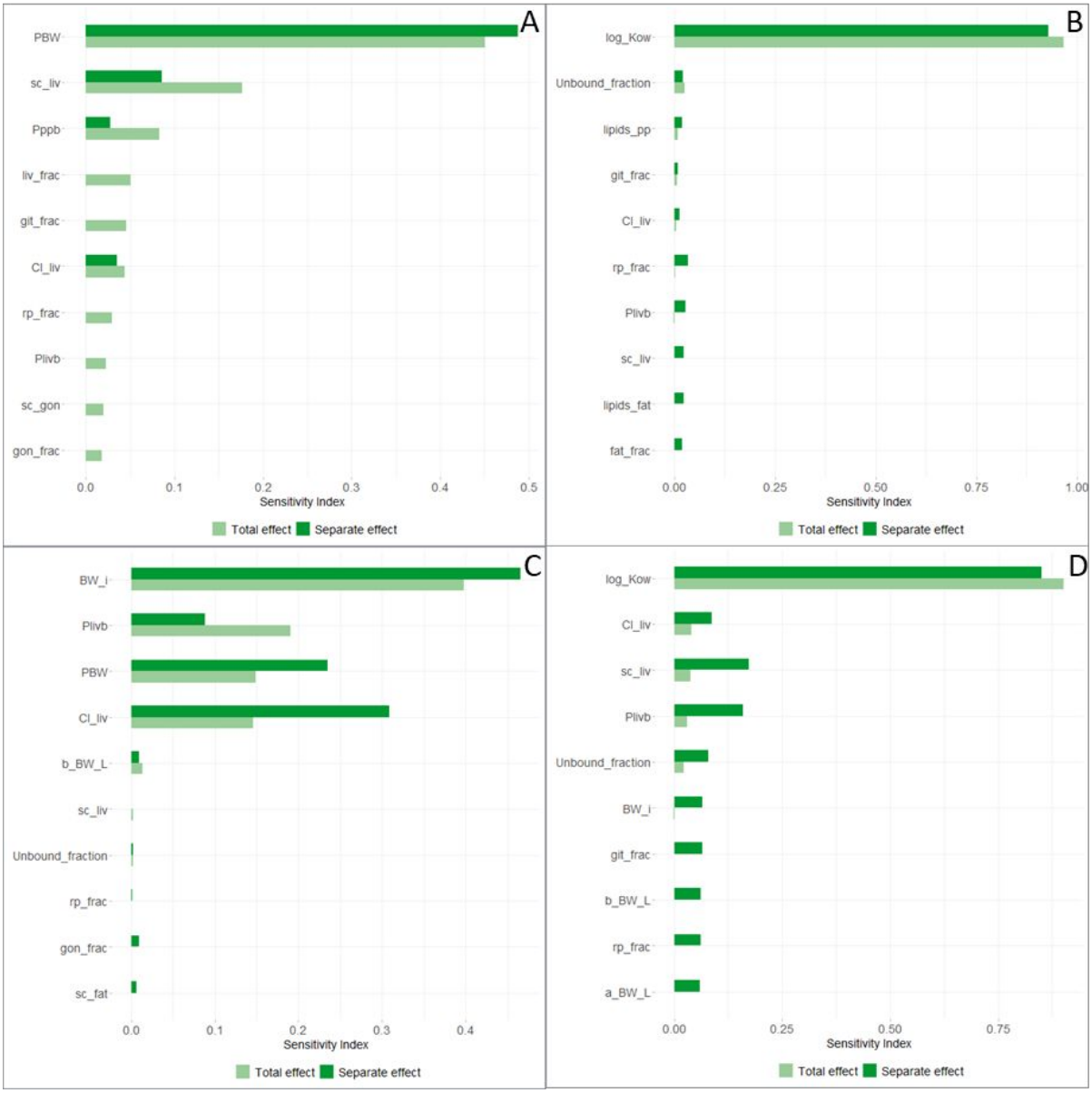

Figure S 6 Sobol indices for the top 10 most influential parameters for BPA model output. Evaluated model outputs are whole body AUC (A and B) and liver AUC (C and D). Analysis was done either by varying the partition coefficient values (A and C) or by using QSPR model for partitioning incorporated in the analysis (B and D)

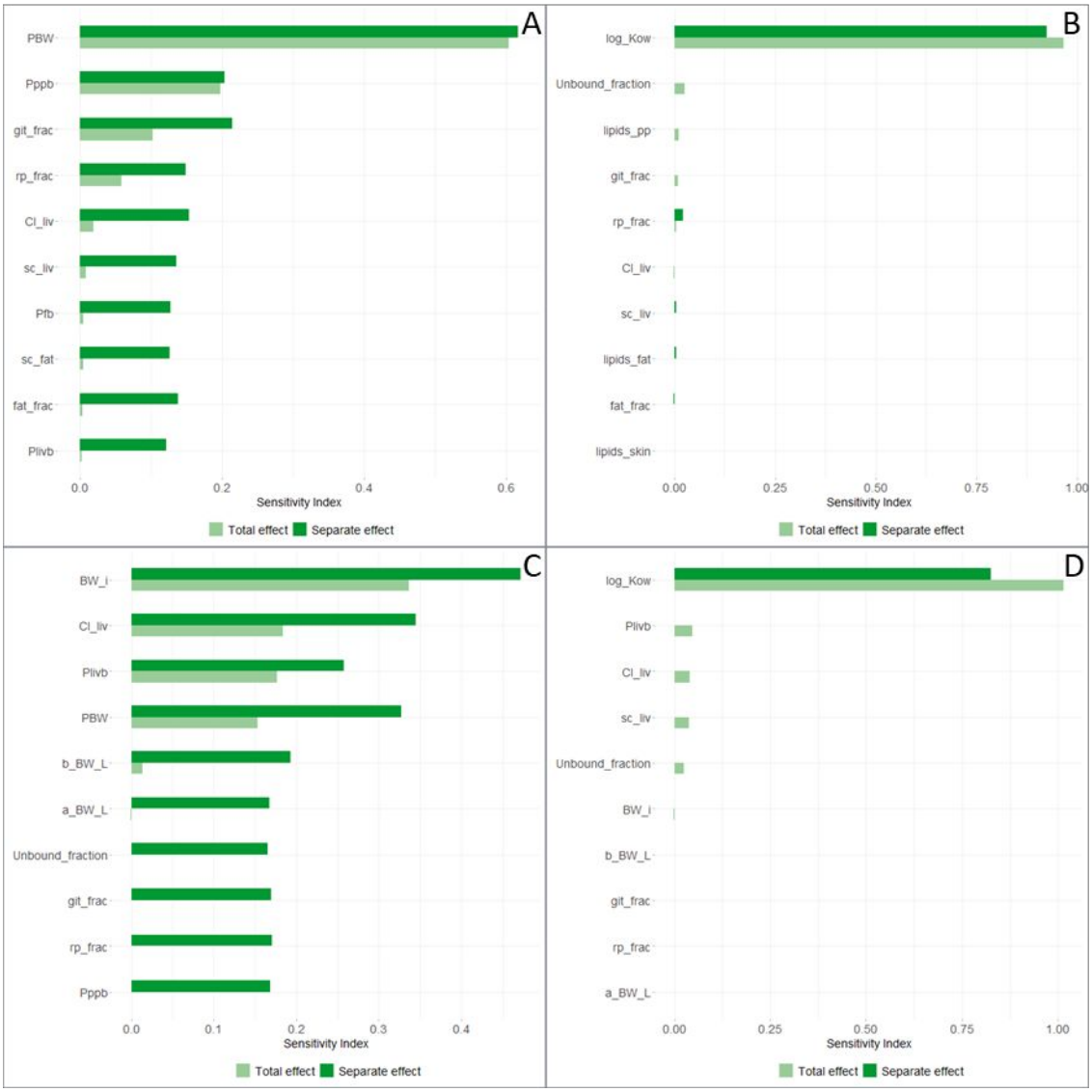

Figure S 7 Sobol indices for the top 10 most influential parameters for BPA model output. Evaluated model outputs are whole body AUC (A and B) and liver AUC (C and D). Analysis was done either by varying the partition coefficient values (A and C) or by using QSPR model for partitioning incorporated in the analysis (B and D)

12. R Script

```
##############################################################################################################
```

Title: Physiologically Based Toxicokinetic Modelling of Bisphenols in Zebrafish (Danio rerio) Accounting for Variation in Metabolic Rates, Brain Distribution and Liver Accumulation

```
### Author: Ioana Chelcea
### Date:   19-11-2021
### Example code for Bisphenol A with data from Chen et al. 2017
###Adapted from:
###Grech, A.; Tebby, C.; Brochot, C.; Bois, F. Y.; Bado-Nilles, A.; Dorne, J.-L.; Quignot, N.; Beaudouin,
R.
###Sci. Total Environ. 2019, 651 (Pt 1), 516–531
### Creative Commons Attribution 4.0 license
### Structure: 1a. PBTK model for parent compound
###         1b. PBTK model for metabolites
###         2. Partition coefficient models
###         3. Output function
###         4. Simulation of events
###         5. Parameter values and simulation run
### Notification: run the entire code
################################################################################
####################


################################
### Variable -(Notation)- Units
library(plyr)
library(dplyr)
library(deSolve)
library(reshape2)
library(httk)
library(MESS)
library(tidyverse)
library(sensitivity)



################################
```

```
### Variable -(Notation)- Units
# Quantity       -(A_x) - microg
# Volumes:      -(V_x) - mL
# Time:          -(t)   - d
# Flows:         -(F_x) - mL/d
# Concentrations: -(C_x) - microg/mL
# Masses:        -(BW)  - g
# Lenght:        -(L)   - mm
# Temperature: -(TC_c)  - Celsius
#     ''              -(TC_k)  - Kelvin
# Density of each tissue is considered equal to 1
################################

#### Arrhenius temperatures function ### as presented by Grech et al. 2019

KT <- function(T, TR, TA){ exp ( (TA / TR) - (TA / T) ) }

####################     PBTK MODEL   ###################


ZF.model <- function(t,initial_v, parameters) {
  with(as.list(c(initial_v, parameters)),{
############################## 1a. PBTK of parent compound ##########################

    dV_egg   = Egggrowth
    PS      = pi^(1/3)*(6*V_one_egg)^(2/3)*(V_egg/V_one_egg)

    DEB_v_t   = DEB_v  * KT(T=TC_k , TR=TR_DEB ,TA=TA)  # mm/d
    DEB_Lm    = DEB_v / (DEB_KM * DEB_g)              # mm
    DEB_M     = (DEB_EHm / DEB_EHb)^(1/3)             # EHm and EHb = J
    dL       = (DEB_v_t / (3 * (f_cst + DEB_g))) * (f_cst * DEB_M - (L/DEB_Lm))
```

```
431     BW      = a_BW_L *( (L/10)/DEB_shape ) ^ b_BW_L   # BW = g; a = g/cm;  L = mm --> /10 = cm
432
433     #Volumes (ml)
434     V_art   = sc_blood  * BW  * art_ven_frac
435     V_ven   = sc_blood  * BW  - V_art
436     V_liv   = sc_liv    * BW
437     V_gon   = sc_gon    * BW
438     V_fat   = sc_fat    * BW
439     V_git   = sc_git    * BW
440     V_brain  = sc_brain  * BW
441     V_kidney = sc_kidney * BW
442     V_skin   = sc_skin   * BW
443     V_rp    = sc_rp     * BW
444     V_pp    = BW-(V_art+V_ven+V_liv+V_gon+V_fat+V_git+V_brain+V_kidney+V_skin+V_rp)
445
446     V_total = V_art+V_ven+V_liv+V_gon+V_fat+V_git+V_brain+V_kidney+V_skin+V_rp+V_pp
447     V_bal = BW- V_total
448
449     #Parameter equations
450     Fcard = (F_card_ref * KT(T=TC_k , TR=TR_Fcard,TA=TA) * (BW/BW_Fcard_ref)^(-0.1))*BW #cardiac
451 output adjusted for temperature and BW of the simulation (ml/d)
452
453     ##Flows to organs (ml/d)
454     Fliv    = liv_frac  * Fcard
455     Fgon    = gon_frac  * Fcard
456     Fgit    = git_frac  * Fcard
457     Ffat            = fat_frac  * Fcard
458     Fbrain  = brain_frac  * Fcard
459     Fkidney = kidney_frac * Fcard
460     Fskin   = skin_frac  * Fcard
461     Frp     = rp_frac     * Fcard
```

```
462     Fpp    = Fcard-(Fliv+Fgon+Fgit+Ffat+Fbrain+Fkidney+Fskin+Frp)
463     Ftotal  = Fliv+Fgon+Fgit+Ffat+Fbrain+Fkidney+Fskin+Frp+Fpp
464     Fbal   = Fcard- Ftotal
465     Fegg   = Fgon
466
467
468
469     VO2    = (VO2_ref*KT(T=TC_k,TR=TR_VO2,TA=TA)*(BW/BW_VO2_ref)^(-0.1))*BW   # O2
470   consumption rate (mg/d/g) adjusted for T and BW of the simmulation
471     Co2w   = ((-0.24 * TC_c + 14.04) * Sat)/10^3                    # (mg O2/mL)C of O2 in water at T in
472   celsius
473     Fwater  = VO2/(OEE*Co2w)                                  #Effective respratory volume. Equation
474   from Barber et al.
475     Cl_plasma_sc   = Cl_plasma*BW
476     if ( !is.na(Vmax))   { Vmax_sc  =  Vmax * V_liv  }
477     if ( !is.na(Cl_liv)) { Cl_sc    = Cl_liv * V_liv }
478
479
480     #####Dosing#####
481     kx = min(Fwater, Fcard * PBW) #the uptake rate is either limited by the respiratory V or by
482   diffusion from water to blood
483
484     #####ABSORPTION#######
485
486     dA_admin_gil = kx * ((A_water)/V_water)
487
488     dA_lumen_GIT = (A_bile* K_BG-Ku*A_lumen_GIT-Ke_feces*A_lumen_GIT)
489
490     dA_admin_git = 0 #A_admin_git
491
492
493     #####ELIMINATION/EXCRETION and METABOLISM#####
```

```
494
495
496     dA_excr_gil = kx * (((A_ven/V_ven)*Unbound_fraction)/PBW)
497
498
499     dV_urine = urine_rate* BW
500     dA_urine = Ke_urine * A_kidney
501     dA_urine_cum = Ke_urine * A_kidney
502
503     dA_feces = A_lumen_GIT * Ke_feces
504
505     dA_excr_water = dA_excr_gil + dA_urine_cum + dA_feces
506
507     dA_bile  = (Ke_bile * A_liv)-(A_bile * K_BG)
508
509
510
511     dA_met_liv    = if ( is.na(Cl_liv))
512     {(Vmax_sc*((A_liv/V_liv)/Plivb))/(Km+(A_liv/V_liv)/Plivb)}
513     else{(Cl_sc*((A_liv/V_liv)/Plivb))}
514
515
516
517     dA_met_plasma = Cl_plasma_sc* A_ven
518
519
520     #Elimination
521
522     dA_Elimination = dA_met_liv+ dA_met_plasma
523
524     #####DISTRIBUTION#####
```

```
525
526     #Blood
527     dA_art   = (Fcard*(A_ven / V_ven)-Fliv*(A_art/V_art)-Ffat*(A_art/V_art)-Fskin*(A_art/V_art)
528             -Fgon*(A_art /V_art)-Fgit*(A_art/V_art)- Fbrain*(A_art/V_art)-Fkidney*(A_art/V_art)
529             -Frp*(A_art /V_art)-Fpp*(A_art /V_art))
530
531
532     dA_ven   = (dA_admin_gil-dA_excr_gil-Fcard*(A_ven/V_ven)+ #The venous compartment is where
533 the chemical gets absorbed
534               +Ffat*((A_fat/V_fat)/Pfb)+a_Fs*Fskin*((A_skin/V_skin)/Pskinb)
535             +Fbrain*((A_brain /V_brain)/Pbb)+(Fliv+Fgon+Fgit+Frp)*((A_liv/V_liv)/Plivb)+
536               +(Fkidney+(1-a_Fs)*Fskin+(1-
537 a_Fpp)*Fpp)*((A_kidney/V_kidney)/Pkidb)+a_Fpp*Fpp*((A_pp/V_pp)/Pppb))
538
539
540     #Organs
541
542     dA_fat   = Ffat*(A_art/V_art)- Ffat* ((A_fat/V_fat)/Pfb)
543
544     dA_skin  = Fskin*(A_art/V_art) - Fskin*((A_skin/V_skin)/Pskinb)
545
546     dA_git   = Ku*A_lumen_GIT + Fgit*(A_art/V_art) - Fgit*((A_git/V_git)/Pgitb)
547
548
549     dA_egg     = PS/V_egg*((A_gon/V_gon)/Pgonb)-PS/V_egg*((A_egg/V_egg)/Pegggon)
550
551     dA_egg_cum  = PS/V_egg*((A_gon/V_gon)/Pgonb)-PS/V_egg*((A_egg/V_egg)/Pegggon)
552
553     dA_gon      = Fgon*(A_art/V_art) - Fgon*((A_gon/V_gon)/Pgonb)-
554 PS/V_egg*((A_gon/V_gon)/Pgonb)+PS/V_egg*((A_egg/V_egg)/Pegggon)
555
556     dA_brain   = Fbrain*(A_art/V_art) - Fbrain*((A_brain/V_brain)/Pbb)
```

```
557
558
559     dA_liv = (Fliv*(A_art/V_art)+ Fgon*((A_gon/V_gon)/Pgonb)+ Fgit*
560 ((A_git/V_git)/Pgitb)+Frp*((A_rp/V_rp)/Prpb)
561             -(Fliv+Fgon+Fgit+Frp)*((A_liv/V_liv)/Plivb)-Ke_bile*A_liv-dA_met_liv)
562
563     dA_kidney = (Fkidney*(A_art/V_art)+(1-a_Fs)*Fskin*((A_skin/V_skin)/Pskinb)+(1-
564 a_Fpp)*Fpp*((A_pp/V_pp)/Pppb)
565               -(Fkidney+(1-a_Fs)*Fskin+(1-a_Fpp)*Fpp)*((A_kidney/V_kidney)/Pkidb) -dA_urine)
566
567     dA_rp    = Frp*(A_art/V_art)- Frp* ((A_rp/V_rp)/Prpb)
568     dA_pp    = Fpp*(A_art/V_art)- Fpp* ((A_pp/V_pp)/Pppb)
569
570     #Water
571     dA_water = (dA_excr_water-dA_admin_gil)
572
573     #####MASS BALANCES#####
574
575     V_tot= V_ven+V_art+V_liv+V_fat+V_gon+V_git+V_brain+V_kidney+V_rp+V_pp+V_skin
576
577     A_body_tot =
578 (A_art+A_ven+A_bile+A_liv+A_fat+A_gon+A_git+A_brain+A_kidney+A_skin+A_rp+A_pp)
579
580     A_input = A_admin_gil+A_admin_git+A_iv
581
582     A_elim = A_met_liv+A_met_plasma+A_excr_water+A_egg_cum+A_lumen_GIT
583
584     A_tot_sys = A_water+A_input+A_body_tot
585
586     Mass_bal_sys = A_input-A_body_tot-A_elim
587
```

```
588
589 ##############################################################################################
590 ############################
591   ############################## 1b. PBTK of METABOLITE
592 ##########################################################################
593
594 ##############################################################################################
595 ############################
596
597 ##############################################################################################
598 ##########################
599
600   #####Dosing#####
601
602   M_kx     = 0    #assumed no Gill excretion
603   M_Vmax_sc =  M_Vmax * V_liv
604
605   #####ABSORPTION#######
606
607   M_dA_admin_gil = M_kx * ((M_A_water)/V_water)
608
609   M_dA_lumen_GIT = (M_A_bile* M_K_BG-M_Ku*M_A_lumen_GIT-M_Ke_feces*M_A_lumen_GIT)
610
611
612   #####ELIMINATION/EXCRETION and METABOLISM#####
613
614
615   M_dA_excr_gil = M_kx * (((M_A_ven/V_ven)*M_Unbound_fraction)/M_PBW)
616
617   M_dA_urine = M_Ke_urine * M_A_kidney
618
619   M_dA_feces = M_A_lumen_GIT * M_Ke_feces
620
621
```

```
622     M_dA_excr_water = M_dA_excr_gil + M_dA_urine + M_dA_feces
623
624     M_dA_bile  = (M_Ke_bile * M_A_liv)-(M_A_bile * M_K_BG)
625
626     M_dA_met_liv = 0
627
628     #Elimination
629
630     M_dA_Elimination = M_dA_met_liv
631
632     #####DISTRIBUTION#####
633
634     #Blood
635     M_dA_art   = (Fcard*(M_A_ven / V_ven)-Fliv*(M_A_art/V_art)-Ffat*(M_A_art/V_art)-
636 Fskin*(M_A_art/V_art)
637             -Fgon*(M_A_art /V_art)-Fgit*(M_A_art/V_art)- Fbrain*(M_A_art/V_art)-
638 Fkidney*(M_A_art/V_art)
639             -Frp*(M_A_art /V_art)-Fpp*(M_A_art /V_art))
640
641
642     M_dA_ven   = (M_dA_admin_gil-M_dA_excr_gil-Fcard*(M_A_ven/V_ven)+
643               +Ffat*((M_A_fat/V_fat)/M_Pfb)+a_Fs*Fskin*((M_A_skin/V_skin)/M_Pskinb)
644             +Fbrain*((M_A_brain /V_brain)/M_Pbb)+(Fliv+Fgon+Fgit+Frp)*((M_A_liv/V_liv)/M_Plivb)+
645               +(Fkidney+(1-a_Fs)*Fskin+(1-a_Fpp)*Fpp)*((M_A_kidney/V_kidney)/M_Pkidb)
646             +a_Fpp*Fpp*((M_A_pp/V_pp)/M_Pppb))
647
648
649     #Organs
650
651     M_dA_fat   = Ffat*(M_A_art/V_art)- Ffat* ((M_A_fat/V_fat)/M_Pfb)
652
653     M_dA_skin  = Fskin*(M_A_art/V_art) - Fskin*((M_A_skin/V_skin)/M_Pskinb)
```

```
654
655     M_dA_git   = M_Ku*M_A_lumen_GIT + Fgit*(M_A_art/V_art) - Fgit*((M_A_git/V_git)/M_Pgitb)
656
657     M_dA_gon   = Fgon*(M_A_art/V_art) - Fgon*((M_A_gon/V_gon)/M_Pgonb)
658
659     M_dA_brain = Fbrain*(M_A_art/V_art) - Fbrain*((M_A_brain/V_brain)/M_Pbb)
660
661
662     M_dA_liv = (Fliv*(M_A_art/V_art)+ Fgon*((M_A_gon/V_gon)/M_Pgonb)+ Fgit*
663 ((M_A_git/V_git)/M_Pgitb)
664             +Frp*((M_A_rp/V_rp)/M_Prpb)-(Fliv+Fgon+Fgit+Frp)*((M_A_liv/V_liv)/M_Plivb)
665             -M_Ke_bile*M_A_liv-M_dA_met_liv+ dA_met_liv)
666
667     M_dA_kidney = (Fkidney*(M_A_art/V_art)+(1-a_Fs)*Fskin*((M_A_skin/V_skin)/M_Pskinb)
668               +(1-a_Fpp)*Fpp*((M_A_pp/V_pp)/M_Pppb)
669               -(Fkidney+(1-a_Fs)*Fskin+(1-a_Fpp)*Fpp)*((M_A_kidney/V_kidney)/M_Pkidb)-
670 M_dA_urine)
671
672     M_dA_rp    = Frp*(M_A_art/V_art)- Frp* ((M_A_rp/V_rp)/M_Prpb)
673     M_dA_pp    = Fpp*(M_A_art/V_art)- Fpp* ((M_A_pp/V_pp)/M_Pppb)
674
675     #Water
676     M_dA_water = (M_dA_excr_water-M_dA_admin_gil)
677
678     #####MASS BALANCES#####
679
680
681     M_A_body_tot =
682 (M_A_art+M_A_ven+M_A_bile+M_A_liv+M_A_fat+M_A_gon+M_A_git+M_A_brain+M_A_kidney+M
683 _A_skin+M_A_rp+M_A_pp)
684
685     M_A_input = M_A_lumen_GIT
```

```
686
687     M_A_tot_sys = M_A_water+M_A_input+ M_A_body_tot
688
689
690     ###CONCENTRATIONS
691
692     C_whole_body = A_body_tot/BW
693     M_C_whole_body = M_A_body_tot/BW
694
695     C_art = A_art/V_art
696     C_ven = A_ven/V_ven
697     C_liv = (A_liv+A_bile)/V_liv
698     C_fat = A_fat/V_fat
699     C_egg = A_egg/V_egg
700     C_gon = A_gon/V_gon
701     C_gon_egg = (A_gon+A_egg)/(V_gon+V_egg)
702     C_git = A_git/V_git
703     C_brain = A_brain/V_brain
704     C_kidney = A_kidney/ V_kidney
705     C_skin = A_skin/V_skin
706     C_rp  = A_rp/V_rp
707     C_pp  = A_pp/V_pp
708     C_carcass = (A_body_tot-A_gon-A_liv-A_brain)/(V_tot-V_gon-V_liv-V_brain)
709
710     M_C_art = M_A_art/V_art
711     M_C_ven = M_A_ven/V_ven
712     M_C_liv = M_A_liv/V_liv
713     M_C_fat = M_A_fat/V_fat
714     M_C_gon = M_A_gon/V_gon
715     M_C_git = M_A_git/V_git
716     M_C_brain = M_A_brain/V_brain
```

```
717       M_C_kidney = M_A_kidney/ V_kidney
718       M_C_skin = M_A_skin/V_skin
719       M_C_rp  = M_A_rp/V_rp
720       M_C_pp  = M_A_pp/V_pp
721
722       list(c(dV_egg,dL, dA_water, dA_excr_water,
723     dA_admin_gil,dA_admin_git,dA_excr_gil,dV_urine,dA_urine, dA_urine_cum,
724              dA_feces,dA_lumen_GIT, dA_met_liv, dA_met_plasma, dA_art, dA_ven, dA_liv,
725              dA_kidney, dA_fat, dA_skin,dA_egg,dA_egg_cum, dA_gon, dA_git, dA_brain, dA_rp,dA_pp,
726              dA_bile, dA_Elimination,
727              M_dA_water, M_dA_excr_water, M_dA_admin_gil,M_dA_excr_gil,M_dA_urine,
728              M_dA_feces,M_dA_lumen_GIT, M_dA_met_liv, M_dA_art, M_dA_ven, M_dA_liv,
729              M_dA_kidney, M_dA_fat, M_dA_skin, M_dA_gon, M_dA_git, M_dA_brain,
730     M_dA_rp,M_dA_pp,
731              M_dA_bile, M_dA_Elimination),
732            "BW"=BW,
733            "A_body_tot"= A_body_tot,
734            "Mass_bal_sys"= Mass_bal_sys,
735            "C_whole_body" = C_whole_body,
736            "M_C_whole_body" = M_C_whole_body,
737            "kx" = kx,
738            "PS" = PS,
739
740            "C_art"= C_art ,
741            "C_ven" = C_ven,
742            "C_liv" = C_liv,
743            "C_fat" = C_fat,
744            "C_egg" = C_egg,
745            "C_gon_egg"= C_gon_egg,
746            "C_gon" = C_gon,
747            "C_git" = C_git,
748            "C_brain" = C_brain,
```

```
749           "C_kidney" = C_kidney,
750           "C_skin" = C_skin,
751           "C_rp" =  C_rp,
752           "C_pp" = C_pp,
753           "C_carcass" = C_carcass
754
755
756         )
757       })}
758
759
760   ################################################################################
761   ################################################################################
762   ##########################
763   ################################################################################
764   ################################################################################
765   ##########################
766   ################################################################################
767   ################################################################################
768   ##########################
769   ############################################################################ 2. PARTITION
770   COEFFICIENT MODELS
771   ################################################################################
772   ######
773   ################################################################################
774   ################################################################################
775   ##########################
776   ################################################################################
777   ################################################################################
778   ##########################
779
780   PC_qsar_model = function(a_PC  = 0.73,
781                            b_PC  = 0.88,
782
783
784                            a_Bar = 0.74, # Parameter 1 of Bertelsen et 1998
```

```
785                 b_Bar = 0.72, # Parameter 2 of Bertelsen et 1998
786                 c_Bar = 1.00, # Parameter 3 of Bertelsen et 1998
787
788                 log_Kow ,
789                 Funbound,
790
791                 water_liver,  water_gonads, water_brain,
792                 water_fat,    water_skin,   water_GIT,
793                 water_kidney, water_rp,     water_pp,
794
795                 lipids_liver, lipids_gonads, lipids_brain,  lipids_fat,
796                 lipids_skin,  lipids_GIT,    lipids_kidney, lipids_rp,
797                 lipids_pp){
798
799
800   #================================================
801   # (I.) QSAR Model : partition coefficient between blood and water,
802   #Fitzsimmons, P. N.; Fernandez, J. D.; Hoffman, A. D.; Butterworth, B. C.; Nichols, J. W.
803   #Aquat. Toxicol. 2001, 55
804   #================================================
805
806   PC_QSAR_blood_water = 10^(a_PC * log_Kow - b_PC)
807
808   PC_blood_water = PC_QSAR_blood_water #2.06
809
810
811
812    #================================================
813    # (II.) QSAR Model
814    # Bertelsen, S. L.; Hoffman, A. D.; Gallinat, C. A.; Elonen, C. M.; Nichols, J. W.
815    # Environ. Toxicol. Chem. 1998, 17, 1447-1455.
```

```
816       #============================================
817
818
819
820       PC_QSAR_liver      = (water_liver   + 10^(a_Bar*log_Kow + b_Bar + c_Bar * log10(lipids_liver ) ))
821     /PC_blood_water
822       PC_QSAR_gonads     = (water_gonads  + 10^(a_Bar*log_Kow + b_Bar + c_Bar *
823     log10(lipids_gonads) )) /PC_blood_water
824       PC_QSAR_brain      = (water_brain   + 10^(a_Bar*log_Kow + b_Bar + c_Bar * log10(lipids_brain)  ))
825     /PC_blood_water
826       PC_QSAR_fat        = (water_fat     + 10^(a_Bar*log_Kow + b_Bar + c_Bar * log10(lipids_fat)    ))
827     /PC_blood_water #2.55
828       PC_QSAR_skin       = (water_skin    + 10^(a_Bar*log_Kow + b_Bar + c_Bar * log10(lipids_skin)   ))
829     /PC_blood_water
830       PC_QSAR_GIT        = (water_GIT     + 10^(a_Bar*log_Kow + b_Bar + c_Bar * log10(lipids_GIT)    ))
831     /PC_blood_water
832       PC_QSAR_kidney     = (water_kidney  + 10^(a_Bar*log_Kow + b_Bar + c_Bar * log10(lipids_kidney)
833     )) /PC_blood_water
834       PC_QSAR_rp         = (water_rp      + 10^(a_Bar*log_Kow + b_Bar + c_Bar * log10(lipids_rp)     ))
835     /PC_blood_water
836       PC_QSAR_pp         = (water_pp      + 10^(a_Bar*log_Kow + b_Bar + c_Bar * log10(lipids_pp)     ))
837     /PC_blood_water
838
839      #============================================
840
841      Plivb    = PC_QSAR_liver
842      Pgonb    = PC_QSAR_gonads
843      Pbb      = PC_QSAR_brain
844      Pfb      = PC_QSAR_fat
845      Pskinb   = PC_QSAR_skin
846      Pgitb    = PC_QSAR_GIT
847      Pkidb    = PC_QSAR_kidney
848      Prpb     = PC_QSAR_rp
849      Pppb     = PC_QSAR_pp
```

```
850     PBW     = PC_blood_water*Funbound #adjust for unbound fraction
851
852     return( c( "PBW"    = PBW, "Pgitb"  = Pgitb,"Pgonb"  = Pgonb,"Plivb"  = Plivb,
853             "Pfb" = Pfb, "Pbb" = Pbb, "Pkidb"=Pkidb,"Pskinb"= Pskinb, "Pppb"= Pppb, "Prpb"= Prpb ))
854   }
855
856
857
858
859
860
861   ##############################################################################################
862   ##############################################################################################
863   ############################
864   ##############################################################################################
865   ##############################################################################################
866   ############################
867   ########################################################################## 3.
868   OUTPUT FUNCTION
869   ##############################################################################################
870   ########
871   ##############################################################################################
872   ##############################################################################################
873   ############################
874   ##############################################################################################
875   ##############################################################################################
876   ############################
877
878
879
880   output <- function(parms){
881
882     #Simulation parameters
883     method = as.character("lsoda") # differential solving method
884     start = parms[["start"]]
```

```
  stop = parms[["stop"]]

  Res_times =  c(0.083, 0.25, 12/24, 24/24, 48/24, 72/24, 120/24, 168/24, 170/24, 7.2, 180/24,
192/24, 216/24, 240/24, 12, 336/24,0.2,0.3,1.0,2.0,3.0,6.0,7.0,8.0,10.0,13.0)# days, measured data

  times = sort( unique( c(seq(start,stop,0.1), Res_times)))#times (days) from 0 to 14 including the
measured data time points


  period = NA      # days between two doses for oral

  frac_renewed    = 0 # fraction of the water of the aquaria renewed

  time_final_dose = parms[["time_final_dose"]] #Chen

  time_first_dose = parms[["time_first_dose"]]

  TC_c  = parms[["TC_c"]] #Water temperature of simulation in Celsisu. Lindholst

  TC_k = TC_c + 273.15 #Water temperature of simulation in Kelvin. Lindholst et al.


  ##INPUTS

  BW_exp = parms[["BW_exp"]]

  BW_i   = ifelse(is.na(BW_exp),parms[["BW_i"]],BW_exp)#0.82  # g, 0.82 for female #0.41 for a male


  A_iv        = parms[["A_iv"]]

  food        = parms[["food"]]

  frac_absorbed = parms[["frac_absorbed"]]

  Dose_water    = parms[["Dose_water"]] #ug/ml

  V_water      = parms[["V_water"]]

  Sat         = 0.9  #dissolved oxygen saturation of 90% proposed by Erickson, 1990 and Hamilton et al
(12 ul/min)


  ##Reference parameters


  TA          = 3000    # Arrhenius temperature  in Kelvin (double check)

  TR_DEB      = 293.65   # (Kelvin)

  f_cst       = 1       # food level 1 = ad-libitum, 0= starvation

  TR_Fcard    = 27 + 273.15  # Reference T for cardiac output (Kelvin), Average of Pery et al 2014 (26)
and Hamilton et al 2014 (28)
```

```
  TR_VO2      = 26 + 273.15  # Reference T for O2 consumption rate (Kelvin) Vergauwen et al

  BW_Fcard_ref = 0.5   #Reference BW for cardiac output value Pery et al 2014 and Hamilton et al
2014

  BW_VO2_ref   = 0.49  # Reference BW for O2 extraction rate value Vergauwen et al

  VO2_ref     = 9.84  # mg O2/d/g --> calculated from ventilation volume proposed by Péry, 2014
(Qw= 0.55 mL/min/g)

  F_card_ref   = 0.5   ##Reference parameters

  TA          = 3000        # Arrhenius temperature  in Kelvin (double check)

  TR_DEB      = 293.65      # (Kelvin)

  f_cst       = 1           # food level 1 = ad-libitum, 0= starvation

  TR_Fcard    = 27 + 273.15  # Reference T for cardiac output (Kelvin), Average of Pery et al 2014  and
Hamilton et al 2014

  TR_VO2      = 26 + 273.15  # Reference T for O2 consumption rate (Kelvin) Vergauwen et al

  BW_Fcard_ref = 0.5   #Reference BW for cardiac output value Pery et al 2014 and Hamilton et al
2014

  BW_VO2_ref   = 0.49  # Reference BW for O2 extraction rate value Vergauwen et al

  VO2_ref     = 9.84  # mg O2/d/g --> calculated from ventilation volume proposed by Péry, 2014
(Qw= 0.55 mL/min/g)

  F_card_ref   = (((11.1+12)/2)*10^(-3))*(24*60)/BW_Fcard_ref #ml/d/g, converted from Pery et al
(11.1 ul/min)

  DEB_v       = 0.6      # Energy conductance (mm/d) (DEB model parameter)

  DEB_g       = 0.427    # Energy investment ratio (SU) (DEB model parameter)

  DEB_KM      = 0.187    # Somatic maintenance rate coefficient (1/d) (DEB model parameter)

  DEB_EHm     = 1        # Energy at State of maturity at metamorphosis (J)

  DEB_EHb     = 1        # Energy at State of maturity at birth (J)

  DEB_shape   = 0.2



  ##### PHYSIOLOGICAL PARAMETERS #####
```

```
950
951     OEE       = parms[["OEE"]]                    #Oxygen extraction efficiency of 71% proposed by Erickson,
952     1990 (Rainbow trout)
953     a_BW_L    = parms[["a_BW_L"]]                 # a relation BW(g)=F(L(cm))
954     b_BW_L    = parms[["b_BW_L"]]                 # b relation BW(g)=F(L(cm))
955     L0        = ((BW_i/ a_BW_L) ^ (1/b_BW_L)) * DEB_shape * 10 # g --> cm Ltotale --> Lstruc --> mm
956
957
958
959     #Blod flow fractions (fraction of cardiac output going to specific organs)
960     liv_frac    = parms[["liv_frac"]]
961     gon_frac    = parms[["gon_frac"]]
962     git_frac    = parms[["git_frac"]]
963     fat_frac    = parms[["fat_frac"]]
964     brain_frac  = parms[["brain_frac"]]
965     art_ven_frac = parms[["art_ven_frac"]]
966     kidney_frac  = parms[["kidney_frac"]]
967     skin_frac   = parms[["skin_frac"]]
968     rp_frac     = parms[["rp_frac"]]
969     pp_frac     = (1- liv_frac- gon_frac- git_frac- fat_frac- brain_frac- kidney_frac- skin_frac-rp_frac )
970     a_Fs        = parms[["a_Fs"]]
971     a_Fpp       = parms[["a_Fpp"]]
972
973     sc_blood  = parms[["sc_blood"]]
974     sc_liv    = parms[["sc_liv"]]
975     sc_gon    = parms[["sc_gon"]]
976     sc_fat    = parms[["sc_fat"]]
977     sc_git    = parms[["sc_git"]]
978     sc_brain  = parms[["sc_brain"]]
979     sc_kidney = parms[["sc_kidney"]]
980     sc_skin   = parms[["sc_skin"]]
```

```
    sc_rp     = parms[["sc_rp"]]
    sc_pp     = (1-sc_blood-sc_liv-sc_gon-sc_fat-sc_git-sc_brain-sc_kidney-sc_skin-sc_rp)

    #####CHEMICAL PARAMETERS#####


    # Chemical parameters
    log_Kow           = parms[["log_Kow"]]
    Unbound_fraction   = parms[["Unbound_fraction"]] # in blood. between 0 and 1. Limits excretion by
gills
    Ratio_blood_plasma = parms[["Ratio_blood_plasma"]] # blood/plasma global equilibrium
(hemtocrite, fu, ...)

    ###  ORGAN COMPOSITION (from Grech et al. 2018)  ####

    water_liver = parms[["water_liver"]]
    water_brain = parms[["water_brain"]]
    water_gonads= parms[["water_gonads"]]
    water_fat   = parms[["water_fat"]]
    water_skin  = parms[["water_skin"]]
    water_GIT   = parms[["water_GIT"]]
    water_kidney= parms[["water_kidney"]]
    water_rp    = parms[["water_rp"]]  # value of zebrafish model
    water_pp    = parms[["water_pp"]]  # muscle value

    lipids_liver = parms[["lipids_liver"]]
    lipids_brain = parms[["lipids_brain"]]
    lipids_gonads= parms[["lipids_gonads"]]
    lipids_fat   = parms[["lipids_fat"]]
    lipids_skin  = parms[["lipids_skin"]]
    lipids_GIT   = parms[["lipids_GIT"]]
    lipids_kidney= parms[["lipids_kidney"]]
```

```
1013      lipids_rp    = parms[["lipids_rp"]]     # value of zebrafish model
1014      lipids_pp    = parms[["lipids_pp"]]     # muscle value
1015
1016
1017
1018      #Excretion#
1019      Ku               = parms[["Ku"]]       #Oral absorption
1020      Ke_urine         = parms[["Ke_urine"]]  #/d
1021      Ke_feces         = parms[["Ke_feces"]]  # 1/d estiamted from Nichols  et al. 2004
1022      urine_rate       = parms[["urine_rate"]]# V_burst = 1.2 mL.kg-1 every 29.82 minutes proposed by
1023    Curtis 1991 --> 1.2e-03 mL.g BW-1
1024      urination_interval = NA                 #No urine excretion
1025
1026      ###SPAWNING###
1027
1028      spawnrate = parms[["spawnrate"]]#days
1029      Egggrowth = parms[["Egggrowth"]]#ml/day (70 eggs/day)
1030      V_egg     = parms[["V_egg"]]    #ml ( based on 0.74mm diameter with assumption of perfect sphere
1031    and density = 1)
1032
1033
1034
1035      PC_BPA  = PC_qsar_model(log_Kow = log_Kow, Funbound = Unbound_fraction,
1036                          water_liver = water_liver  , lipids_liver  = lipids_liver ,
1037                          water_gonads= water_gonads , lipids_gonads = lipids_gonads,
1038                          water_brain = water_brain  , lipids_brain  = lipids_brain,
1039                          water_fat   = water_fat    , lipids_fat    = lipids_fat,
1040                          water_skin  = water_skin   , lipids_skin   = lipids_skin,
1041                          water_GIT   = water_GIT    , lipids_GIT    = lipids_GIT,
1042                          water_kidney= water_kidney , lipids_kidney = lipids_kidney,
1043                          water_rp    = water_rp     , lipids_rp     = lipids_rp,
1044                          water_pp    = water_pp     ,  lipids_pp     = lipids_pp)
```

```
1045
1046
1047
1048
1049  ##############################################METABOLISM#############################
1050  ########################
1051
1052    M_log_Kow = parms[["M_log_Kow"]]
1053
1054    M_Unbound_fraction = 1 #Default
1055
1056
1057
1058    PC_BPAGA = PC_qsar_model(log_Kow = M_log_Kow, Funbound = M_Unbound_fraction,
1059                      water_liver = water_liver  , lipids_liver  = lipids_liver ,
1060                      water_gonads= water_gonads , lipids_gonads = lipids_gonads,
1061                      water_brain = water_brain  , lipids_brain  = lipids_brain,
1062                      water_fat   = water_fat    , lipids_fat    = lipids_fat,
1063                      water_skin  = water_skin   , lipids_skin   = lipids_skin,
1064                      water_GIT   = water_GIT    , lipids_GIT    = lipids_GIT,
1065                      water_kidney= water_kidney , lipids_kidney = lipids_kidney,
1066                      water_rp    = water_rp     , lipids_rp     = lipids_rp,
1067                      water_pp    = water_pp     ,  lipids_pp    = lipids_pp)
1068
1069    names(PC_BPAGA)<-c("PBW"    = "M_PBW", "Pgitb"  = "M_Pgitb","Pgonb"  = "M_Pgonb","Plivb"  =
1070  "M_Plivb",
1071                  "Pfb" = "M_Pfb", "Pbb" = "M_Pbb", "Pkidb"="M_Pkidb",
1072                  "Pskinb"= "M_Pskinb", "Pppb"= "M_Pppb", "Prpb"= "M_Prpb")
1073
1074    #Excretion#
1075    M_Ke_urine = parms[["M_Ke_urine"]]  #/d #visually fitted value assuming BPAGA is only eliminated
1076  via urine
```

```
1077        M_Ke_feces = 0.83
1078        M_Ke_bile  = parms[["M_Ke_bile"]]    # 1/d chemical rate constant from liver to bile.
1079        M_K_BG     = parms[["M_K_BG"]]     # 1/d chemical rate constant from liver to bile.
1080        M_Ku       = parms[["M_Ku"]]    # 1/d Oral absorption
1081
1082        M_Vmax = 0         #ug/d/g liver
1083        M_Km   = 0         #ug/ml
1084
1085
1086
1087        parameters = c(## EXPERIMENTAL CONDITIONS ##
1088          Dose_water   = Dose_water,
1089          A_iv         = A_iv,
1090          food         = food,
1091          V_water      = V_water,
1092          BW_i         = BW_i,
1093          TC_c         = TC_c ,
1094          TC_k         = TC_k ,
1095
1096          ## GROWTH AND TEMPERATURE MODELS
1097          TA           = TA   ,
1098          TR_DEB       = TR_DEB,
1099          f_cst        = f_cst,
1100          TR_Fcard     = TR_Fcard,
1101          TR_VO2       = TR_VO2,
1102          BW_Fcard_ref = BW_Fcard_ref,
1103          BW_VO2_ref   = BW_VO2_ref,
1104          VO2_ref      = VO2_ref,
1105          DEB_v        = DEB_v,
1106          DEB_g        = DEB_g,
1107          DEB_KM       = DEB_KM,
```

```
1108        DEB_EHm     = DEB_EHm,
1109        DEB_EHb     = DEB_EHb,
1110        DEB_shape   = DEB_shape,
1111        a_BW_L      = a_BW_L,
1112        b_BW_L      = b_BW_L,
1113        Sat         = Sat,
1114        F_card_ref  = F_card_ref,
1115        L0          = L0,
1116
1117        ## PHYSIOLOGICAL PARAMETERS ##
1118        OEE         = parms[["OEE"]],
1119
1120        liv_frac    = parms[["liv_frac"]],
1121        gon_frac    = parms[["gon_frac"]],
1122        git_frac    = parms[["git_frac"]],
1123        fat_frac    = parms[["fat_frac"]],
1124        brain_frac  = parms[["brain_frac"]],
1125        art_ven_frac = parms[["art_ven_frac"]],
1126        kidney_frac  = parms[["kidney_frac"]],
1127        skin_frac   = parms[["skin_frac"]],
1128        rp_frac     = parms[["rp_frac"]],
1129        pp_frac     = parms[["pp_frac"]],
1130        a_Fs        = parms[["a_Fs"]],
1131        a_Fpp       = parms[["a_Fpp"]],
1132
1133        sc_blood  = parms[["sc_blood"]],
1134        sc_liv    = parms[["sc_liv"]],
1135        sc_gon    = parms[["sc_gon"]],
1136        sc_fat    = parms[["sc_fat"]],
1137        sc_git    = parms[["sc_git"]],
1138        sc_brain  = parms[["sc_brain"]],
```

```
1139        sc_kidney = parms[["sc_kidney"]],
1140        sc_skin   = parms[["sc_skin"]],
1141        sc_rp     = parms[["sc_rp"]],
1142        sc_pp     = parms[["sc_pp"]],
1143
1144        spawnrate         = parms[["spawnrate"]],
1145        Egggrowth         = parms[["Egggrowth"]],
1146        V_one_egg         = parms[["V_egg"]],
1147
1148        ## CHEMICAL PARAMETERS  ##
1149        Ku                = parms[["Ku"]],
1150        Ke_urine          = parms[["Ke_urine"]],
1151        Ke_feces          = parms[["Ke_feces"]],
1152        Ke_bile           = parms[["Ke_bile"]], #dummy variable
1153        K_BG              = parms[["K_BG"]],    #dummy variable
1154        urine_rate        = parms[["urine_rate"]],
1155        urination_interval = parms[["urination_interval"]],
1156        Cl_liv            = parms[["Cl_liv"]],
1157        Vmax              = parms[["Vmax"]],
1158        Km                = parms[["Km"]],
1159        Cl_plasma         = parms[["Cl_plasma"]],
1160        frac_absorbed     = parms[["frac_absorbed"]],
1161        log_Kow           = parms[["log_Kow"]],
1162        Unbound_fraction   = parms[["Unbound_fraction"]],
1163        Ratio_blood_plasma = parms[["Ratio_blood_plasma"]],
1164
1165        ## PARTITION COEFFICIENTS ##
1166        PBW     = PC_BPA[["PBW"]],#parms[["PBW"]],
1167        Plivb   = parms[["Plivb"]],
1168        Pgonb   = PC_BPA[["Pgonb"]],
1169        Pbb     = parms[["Pbb"]],
```

```
1170        Pfb     = PC_BPA[["Pfb"]],
1171        Pskinb  = PC_BPA[["Pskinb"]],
1172        Pgitb   = PC_BPA[["Pgitb"]],
1173        Pkidb   = PC_BPA[["Pkidb"]],
1174        Prpb    = PC_BPA[["Prpb"]],
1175        Pppb    = PC_BPA[["Pppb"]],
1176        Pegggon = parms[["Pegggon"]],
1177
1178        ## METABOLITE ##
1179        M_log_Kow = parms[["M_log_Kow"]],
1180        M_Unbound_fraction = M_Unbound_fraction,
1181        M_Ke_urine = parms[["M_Ke_urine"]],
1182        M_Ke_feces = M_Ke_feces,
1183        M_Ke_bile  = M_Ke_bile,
1184        M_K_BG     = M_K_BG,
1185        M_Ku       = M_Ku,
1186        M_Vmax     = M_Vmax,
1187        M_Km       = M_Km,
1188
1189        {PC_BPAGA = PC_BPAGA}
1190
1191      )
1192
1193
1194      #Initial conditions
1195      initial_v = c(V_egg         = V_egg,
1196                    L             = L0,
1197                    A_water       = (Dose_water*V_water),
1198                    A_excr_water  = 0,
1199                    A_admin_gil   = 0,
1200                    A_admin_git   = (food*frac_absorbed),
```

```
1201            A_excr_gil    = 0,
1202            V_urine       = 0,
1203            A_urine       = 0,
1204            A_urine_cum   = 0,
1205            A_feces       = 0,
1206            A_lumen_GIT   = (food*frac_absorbed),
1207            A_met_liv     = 0,
1208            A_met_plasma  = 0,
1209            A_art         = 0,
1210            A_ven         = A_iv,
1211            A_liv         = 0,
1212            A_kidney      = 0,
1213            A_fat         = 0,
1214            A_skin        = 0,
1215            A_egg         = 0,
1216            A_egg_cum     = 0,
1217            A_gon         = 0,
1218            A_git         = 0,
1219            A_brain       = 0,
1220            A_rp          = 0,
1221            A_pp          = 0,
1222            A_bile        = 0,
1223            Elimination   = 0,
1224            M_A_water     = 0,
1225            M_A_excr_water = 0,
1226            M_A_admin_gil  = 0,
1227            M_A_excr_gil   = 0,
1228            M_A_urine     = 0,
1229            M_A_feces     = 0,
1230            M_A_lumen_GIT  = 0,
1231            M_A_met_liv   = 0,
```

```
            M_A_art       = 0,
            M_A_ven       = 0,
            M_A_liv       = 0,
            M_A_kidney    = 0,
            M_A_fat       = 0,
            M_A_skin      = 0,
            M_A_gon       = 0,
            M_A_git       = 0,
            M_A_brain     = 0,
            M_A_rp        = 0,
            M_A_pp        = 0,
            M_A_bile      = 0,
            M_Elimination  = 0)



##############################################################################################
##############################################################################################
##########################

##############################################################################################
##############################################################################################
##########################

 ############################################## 4.   EVENTS
##############################################################################################
########

##############################################################################################
##############################################################################################
##########################

##############################################################################################
```

```
1267  ##############################################################################################
1268  ##########################
1269
1270
1271   ########################################  URINE
1272  ####################################################
1273   if (!is.na(urination_interval)){
1274    events_urine <- list(data = rbind(data.frame(var = c("V_urine"),
1275                                  time = seq(times[1], rev(times)[1] , by=urination_interval),
1276                                  value = 0,
1277                                  method = c("replace")),
1278                           data.frame(var = c("A_urine"),
1279                                  time = seq(times[1], rev(times)[1] , by=urination_interval),
1280                                  value = 0,
1281                                  method = c("replace"))))
1282   }else{ events_urine <- NULL }
1283
1284
1285   #=============================================
1286   #(II.3) Exposure scenario :   events_repeated
1287   #=============================================
1288   events_repeated_f <- if (!is.null(period) & food!=0 ) {
1289
1290    # no stop before the end of the experiement
1291    if (is.null(time_final_dose)){ time_final_dose <- floor(max(times)/period)*period}
1292
1293    # First dose NOT at t0 of the experiment
1294    if (!is.null(time_first_dose)) { initial_v["A_admin_git"]<-0 ; initial_v["A_lumen_GIT"]<-0 }
1295
1296    # First dose at t0 of the experiment
1297    if (is.null(time_first_dose)){ time_first_dose = period }
1298
```

```
    # events : a list with a data frame for each compartment --> Cpt name / Time / Dose / method
    events_repeated_f <- list(data = rbind(data.frame(var = c("A_lumen_GIT"),
                                      time = seq(time_first_dose, time_final_dose , by=period),
                                      value =
as.numeric(c(parameters["frac_absorbed"]*parameters["food"])),
                                      method = c("add")),
                           data.frame(var = c("A_admin_git"),
                                      time = seq(time_first_dose, time_final_dose , by=period),
                                      value =
as.numeric(c(parameters["frac_absorbed"]*parameters["food"])),
                                      method = c("add")))) # uses Initial.Values for the first bolus
   }


   events_repeated_w <- NULL


   if (!is.na(period) & Dose_water!=0 ) {

    # no stop before the end of the experiement
    if (is.null(time_final_dose)){ time_final_dose <- floor(max(times)/period)*period}

    # First dose NOT at t0 of the experiment
    if (!is.null(time_first_dose)) { initial_v["A_water"]<-0 }

    # First dose at t0 of the experiment
    if (is.null(time_first_dose)){ time_first_dose = period }

    # events : a list with a data frame for each compartment --> Cpt name / Time / Dose / method
    events_repeated_w <- list(data = rbind(data.frame(var = c("A_water"),
                                      time  = seq(time_first_dose, time_final_dose , by=period),
                                      value = (1-frac_renewed),
```

```
                                    method = c("multiply")),
                               data.frame(var = c("A_water"),
                                    time =  0.0000001+seq(time_first_dose, time_final_dose , by=period),
                                    value = as.numeric( parameters["Dose_water"]* frac_renewed),
                                    method = c("add")),
                               data.frame(var = c("A_water"),
                                    time  = time_final_dose,
                                    value = 0,
                                    method = c("multiply"))

                               # In depuration phase, water renewal
                               #  ,    data.frame(var = c("A_water"),
                               #           time  = 0.0000001+seq(time_final_dose, stop , by=period),
                               #           value = (1-frac_renewed),
                               #           method = c("multiply"))
     ))
    }

    if ((is.na(period))& (Dose_water!=0) ){
      events_repeated_w <- list(data = rbind(data.frame(var = c("A_water"),
                                    time  = time_final_dose,
                                    value = 0,
                                    method = c("multiply"))))
    }


    events_spawn <- list (data = rbind(data.frame(var = c("A_egg"),
                                  time  = seq(spawnrate,stop,spawnrate),
                                  value = 0,
```

```
                                method = c("replace")),
                        data.frame(var = c("V_egg"),
                                time  = seq(spawnrate,stop,spawnrate),
                                value = 2.12E-04,
                                method = c("replace"))
  ))



  events<-list(data = rbind(events_repeated_f[[1]], events_repeated_w[[1]], events_urine[[1]],
events_spawn[[1]]))


  ## solve ##
  solution <- ode(times = times, y = initial_v, func = ZF.model, parms = parameters, method = method,
events = events)


  return(solution)
}


##############################################################################
##############################################################################
#########################

##############################################################################
##############################################################################
#########################
```

```
1395  ######################################################################## 5.
1396  PARAMETERS
1397  ##########################################################################################
1398  ########

1399  ##########################################################################################
1400  ##########################################################################################
1401  ##########################

1402  ##########################################################################################
1403  ##########################################################################################
1404  ##########################

1405  parms= c(

1406   start = 0,

1407   stop = 14,

1408   time_final_dose = 6, #Chen et al.2017

1409   time_first_dose = 0,

1410   TC_c  = 25,

1411   A_iv        = 0,

1412   food        = 0,

1413   Dose_water    = 2 * 10^(-3), #ug/ml = 2 ug/l Chen et al

1414   V_water      = 10E12, # Water compartment is set very large to account for flow through and make
1415  changes in water c insignificant. Like suggested by Remy Beaudouin #aquarium size in ml --> 100L

1416   BW_exp = 1,

1417   BW_i          =          0.82          ,

1418   liv_frac      =          0.02          ,

1419   gon_frac      =          0.01          ,

1420   git_frac      =          0.17          ,

1421   fat_frac      =          0.01          ,

1422   brain_frac    =          0.03          ,

1423   kidney_frac   =          0.02          ,

1424   skin_frac     =          0.06          ,

1425   rp_frac       =          0.13          ,

1426   pp_frac       =          0.55          ,

1427   art_ven_frac =           0.33          ,

1428   Ratio_blood_plasma       =          1.08          ,
```

```
a_Fs = 0.1 ,
a_Fpp = 0.4 ,
sc_blood = 0.02 ,
sc_liv = 0.03 ,
sc_gon = 0.08 ,
sc_fat = 0.02 ,
sc_git = 0.1 ,
sc_brain = 0.008 ,
sc_kidney = 0.002 ,
sc_skin = 0.1 ,
sc_rp = 0.03 ,
sc_pp = 0.61 ,
spawnrate = 1.5 ,
Egggrowth = 0.027 ,
V_egg = 2.12E-04 ,
urine_rate = 0.058 ,
urination_interval = NA ,
a_BW_L = 0.0089 ,
b_BW_L = 3.2355 ,
OEE = 0.71 ,
water_liver = 0.69 ,
water_brain = 0.76 ,
water_gonads = 0.65 ,
water_fat = 0.03 ,
water_skin = 0.76 ,
water_GIT = 0.62 ,
water_kidney = 0.49 ,
water_rp = 0.5293 ,
water_pp = 0.69 ,
lipids_liver = 0.07 ,
lipids_brain = 0.09 ,
```

```
1460    lipids_gonads           =       0.08        ,
1461    lipids_fat      =       1           ,
1462    lipids_skin     =       0.05        ,
1463    lipids_GIT      =       0.04        ,
1464    lipids_kidney   =       0.17        ,
1465    lipids_rp       =       0.07        ,
1466    lipids_pp       =       0.06        ,
1467    Ku              =       0           ,
1468    Ke_urine        =       0           ,
1469    Ke_feces        =       0           ,
1470    Ke_bile         =       0           ,
1471    K_BG            =       1E12        ,
1472    Cl_liv          =       2449        ,
1473    Vmax            =       0           ,
1474    Km              =       0           ,
1475    Cl_plasma       =       0           ,
1476    frac_absorbed           =       1           ,
1477    log_Kow         =       3.42        ,
1478    Unbound_fraction        =       0.035       ,
1479    Plivb           =       195 ,  # fitted on BPA data
1480    Pbb             =       0.42        ,#fitted based on BPZ
1481    Pegggon         =       1.49        ,#fitted based on TBBPA
1482    M_log_Kow       =       2.1         ,#Predicted by Episuite
1483    M_Wsol          =       793.6       ,
1484    M_Ke_bile  = 12.665,    # 1/d chemical rate constant from liver to bile. Fitted based on BPA-GA
1485    M_K_BG     = 0.7,    # 1/d chemical rate constant from bile to gut.Fitted based on BPA-GA
1486    M_Ku       = 0 ,
1487    M_Ke_urine  =           0)
1488
1489  modeloutput<- output(parms)
1490
```

13. References

(1) *Kemikalieinspektionen*. https://www.kemi.se/ (accessed 2019-01-15).

(2) *Rapport 5/17: Bisfenoler – en kartläggning och analys*. https://beta.kemi.se/en/publications/reports/2017/rapport-5-17-bisfenoler---en-kartlaggning-och-analys (accessed 2020-09-28).

(3) Liao, C.; Kannan, K. A Survey of Alkylphenols, Bisphenols, and Triclosan in Personal Care Products from China and the United States. *Arch. Environ. Contam. Toxicol.* **2014**, *67* (1), 50–59. https://doi.org/10.1007/s00244-014-0016-8.

(4) *SciFinder.cas.org*. https://scifinder.cas.org/scifinder/login?TYPE=33554433&REALMOID=06-b7b15cf0-642b-1005-963a-830c809fff21&GUID=&SMAUTHREASON=0&METHOD=GET&SMAGENTNAME=-SM-8iKaCGmTCGHP7yOOI24GDUsJNzy%2bOcz79s1IdZR3o%2fpMdGZxHUbYH371HFTEMp2Z&TARGET=-SM-http%3a%2f%2fscifinder%2ecas%2eorg%3a443%2fscifinder%2f (accessed 2019-03-19).

(5) Chen, D.; Kannan, K.; Tan, H.; Zheng, Z.; Feng, Y.-L.; Wu, Y.; Widelka, M. Bisphenol Analogues Other Than BPA: Environmental Occurrence, Human Exposure, and Toxicity—A Review. *Environ. Sci. Technol.* **2016**, *50* (11), 5438–5453. https://doi.org/10.1021/acs.est.5b05387.

(6) Bhandari, R. K.; Deem, S. L.; Holliday, D. K.; Jandegian, C. M.; Kassotis, C. D.; Nagel, S. C.; Tillitt, D. E.; vom Saal, F. S.; Rosenfeld, C. S. Effects of the Environmental Estrogenic Contaminants Bisphenol A and 17α-Ethinyl Estradiol on Sexual Development and Adult Behaviors in Aquatic Wildlife Species. *Gen. Comp. Endocrinol.* **2015**, *214*, 195–219. https://doi.org/10.1016/j.ygcen.2014.09.014.

(7) Tišler, T.; Krel, A.; Gerželj, U.; Erjavec, B.; Dolenc, M. S.; Pintar, A. Hazard Identification and Risk Characterization of Bisphenols A, F and AF to Aquatic Organisms. *Environ. Pollut. Barking Essex 1987* **2016**, *212*, 472–479. https://doi.org/10.1016/j.envpol.2016.02.045.

(8) Lee, S.; Liao, C.; Song, G.-J.; Ra, K.; Kannan, K.; Moon, H.-B. Emission of Bisphenol Analogues Including Bisphenol A and Bisphenol F from Wastewater Treatment Plants in Korea. *Chemosphere* **2015**, *119*, 1000–1006. https://doi.org/10.1016/j.chemosphere.2014.09.011.

(9) Crain, D. A.; Eriksen, M.; Iguchi, T.; Jobling, S.; Laufer, H.; LeBlanc, G. A.; Guillette, L. J. An Ecological Assessment of Bisphenol-A: Evidence from Comparative Biology. *Reprod. Toxicol.* **2007**, *24* (2), 225–239. https://doi.org/10.1016/j.reprotox.2007.05.008.

(10) *Tox21 Data*. https://tripod.nih.gov/tox21/assays/ (accessed 2020-11-10).

(11) US EPA, O. *Download EPI Suite™ - Estimation Program Interface v4.11*. US EPA. https://www.epa.gov/tsca-screening-tools/download-epi-suitetm-estimation-program-interface-v411 (accessed 2019-01-14).

(12) *Jchem for Office -Chemaxon*. https://chemaxon.com/products/jchem-for-office (accessed 2019-10-29).

(13) Williams, A. J.; Grulke, C. M.; Edwards, J.; McEachran, A. D.; Mansouri, K.; Baker, N. C.; Patlewicz, G.; Shah, I.; Wambaugh, J. F.; Judson, R. S.; Richard, A. M. The CompTox Chemistry Dashboard: A Community Data Resource for Environmental Chemistry. *J. Cheminformatics* **2017**, *9* (1), 61. https://doi.org/10.1186/s13321-017-0247-6.

(14) Weisbrod, C. J.; Kunz, P. Y.; Zenker, A. K.; Fent, K. Effects of the UV Filter Benzophenone-2 on Reproduction in Fish. *Toxicol. Appl. Pharmacol.* **2007**, *225* (3), 255–266. https://doi.org/10.1016/j.taap.2007.08.004.

(15) Liu, K.; Li, J.; Yan, S.; Zhang, W.; Li, Y.; Han, D. A Review of Status of Tetrabromobisphenol A (TBBPA) in China. *Chemosphere* **2016**, *148*, 8–20. https://doi.org/10.1016/j.chemosphere.2016.01.023.

(16) Liu, J.; Zhang, L.; Lu, G.; Jiang, R.; Yan, Z.; Li, Y. Occurrence, Toxicity and Ecological Risk of Bisphenol A Analogues in Aquatic Environment – A Review. *Ecotoxicol. Environ. Saf.* **2021**, *208*, 111481. https://doi.org/10.1016/j.ecoenv.2020.111481.
(17) Mao, F.; He, Y.; Yew-Hoong Gin, K. Occurrence and Fate of Benzophenone-Type UV Filters in Aquatic Environments: A Review. *Environ. Sci. Water Res. Technol.* **2019**, *5* (2), 209–223. https://doi.org/10.1039/C8EW00539G.
(18) Owczarek, K.; Kubica, P.; Kudłak, B.; Rutkowska, A.; Konieczna, A.; Rachoń, D.; Namieśnik, J.; Wasik, A. Determination of Trace Levels of Eleven Bisphenol A Analogues in Human Blood Serum by High Performance Liquid Chromatography–Tandem Mass Spectrometry. *Sci. Total Environ.* **2018**, *628–629*, 1362–1368. https://doi.org/10.1016/j.scitotenv.2018.02.148.
(19) Morris, S.; Allchin, C. R.; Zegers, B. N.; Haftka, J. J. H.; Boon, J. P.; Belpaire, C.; Leonards, P. E. G.; van Leeuwen, S. P. J.; de Boer, J. Distribution and Fate of HBCD and TBBPA Brominated Flame Retardants in North Sea Estuaries and Aquatic Food Webs. *Environ. Sci. Technol.* **2004**, *38* (21), 5497–5504. https://doi.org/10.1021/es049640i.
(20) Kitamura, S.; Suzuki, T.; Sanoh, S.; Kohta, R.; Jinno, N.; Sugihara, K.; Yoshihara, S.; Fujimoto, N.; Watanabe, H.; Ohta, S. Comparative Study of the Endocrine-Disrupting Activity of Bisphenol A and 19 Related Compounds. *Toxicol. Sci.* **2005**, *84* (2), 249–259. https://doi.org/10.1093/toxsci/kfi074.
(21) PubChem. *1,1-Bis(4-hydroxyphenyl)cyclohexane*. https://pubchem.ncbi.nlm.nih.gov/compound/232446 (accessed 2021-10-27).
(22) Chen, F.; Gong, Z.; Kelly, B. C. Bioaccumulation Behavior of Pharmaceuticals and Personal Care Products in Adult Zebrafish (Danio Rerio): Influence of Physical-Chemical Properties and Biotransformation. *Environ. Sci. Technol.* **2017**, *51* (19), 11085–11095. https://doi.org/10.1021/acs.est.7b02918.
(23) Shi, J.; Jiao, Z.; Zheng, S.; Li, M.; Zhang, J.; Feng, Y.; Yin, J.; Shao, B. Long-Term Effects of Bisphenol AF (BPAF) on Hormonal Balance and Genes of Hypothalamus-Pituitary-Gonad Axis and Liver of Zebrafish (Danio Rerio), and the Impact on Offspring. *Chemosphere* **2015**, *128*, 252–257. https://doi.org/10.1016/j.chemosphere.2015.01.060.
(24) Lindholst, C.; Wynne, P. M.; Marriott, P.; Pedersen, S. N.; Bjerregaard, P. Metabolism of Bisphenol A in Zebrafish (Danio Rerio) and Rainbow Trout (Oncorhynchus Mykiss) in Relation to Estrogenic Response. *Comp. Biochem. Physiol. Part C Toxicol. Pharmacol.* **2003**, *135* (2), 169–177. https://doi.org/10.1016/S1532-0456(03)00088-7.
(25) Fang, Q.; Shi, Q.; Guo, Y.; Hua, J.; Wang, X.; Zhou, B. Enhanced Bioconcentration of Bisphenol A in the Presence of Nano-TiO2 Can Lead to Adverse Reproductive Outcomes in Zebrafish. *Environ. Sci. Technol.* **2016**, *50* (2), 1005–1013. https://doi.org/10.1021/acs.est.5b05024.
(26) Shi, J.; Yang, Y.; Zhang, J.; Feng, Y.; Shao, B. Uptake, Depuration and Bioconcentration of Bisphenol AF (BPAF) in Whole-Body and Tissues of Zebrafish (Danio Rerio). *Ecotoxicol. Environ. Saf.* **2016**, *132*, 339–344. https://doi.org/10.1016/j.ecoenv.2016.05.025.
(27) Nyholm, J. R.; Norman, A.; Norrgren, L.; Haglund, P.; Andersson, P. L. Uptake and Biotransformation of Structurally Diverse Brominated Flame Retardants in Zebrafish (Danio Rerio) after Dietary Exposure. *Environ. Toxicol. Chem.* **2009**, *28* (5), 1035–1042. https://doi.org/10.1897/08-302.1.
(28) Tocher, D. R.; Agaba, M.; Hastings, N.; Bell, J. G.; Dick, J. R.; Teale, A. J. Nutritional Regulation of Hepatocyte Fatty Acid Desaturation and Polyunsaturated Fatty Acid Composition in Zebrafish (Danio Rerio) and Tilapia (Oreochromis Niloticus). *Fish Physiol. Biochem.* **2001**, *24* (4), 309–320. https://doi.org/10.1023/A:1015022406790.
(29) Grech, A.; Tebby, C.; Brochot, C.; Bois, F. Y.; Bado-Nilles, A.; Dorne, J.-L.; Quignot, N.; Beaudouin, R. Generic Physiologically-Based Toxicokinetic Modelling for Fish: Integration of Environmental Factors and Species Variability. *Sci. Total Environ.* **2019**, *651* (Pt 1), 516–531. https://doi.org/10.1016/j.scitotenv.2018.09.163.

(30) Barron, M. G.; Tarr, B. D.; Hayton, W. L. Temperature-Dependence of Cardiac Output and Regional Blood Flow in Rainbow Trout, Salmo Gairdneri Richardson. *J. Fish Biol.* **1987**, *31* (6), 735–744. https://doi.org/10.1111/j.1095-8649.1987.tb05276.x.
(31) Wood, C. M.; Shelton, G. Cardiovascular Dynamics and Adrenergic Responses of the Rainbow Trout in Vivo. *J. Exp. Biol.* **1980**, *87* (1), 247–270.
(32) Kooijman, A. A. L. M. Notation of Dynamic Energy Budget Theory for Metabolic Organisation; Cambridge University Press, 2010.
(33) Bertelsen, S. L.; Hoffman, A. D.; Gallinat, C. A.; Elonen, C. M.; Nichols, J. W. Evaluation of Log KOW and Tissue Lipid Content as Predictors of Chemical Partitioning to Fish Tissues. *Environ. Toxicol. Chem.* **1998**, *17* (8), 1447–1455. https://doi.org/10.1002/etc.5620170803.
(34) Larsson, D. G. J.; Adolfsson-Erici, M.; Parkkonen, J.; Pettersson, M.; Berg, A. H.; Olsson, P.-E.; Förlin, L. Ethinyloestradiol — an Undesired Fish Contraceptive? *Aquat. Toxicol.* **1999**, *45* (2), 91–97. https://doi.org/10.1016/S0166-445X(98)00112-X.
(35) Yang, J.; Li, H.; Ran, Y.; Chan, K. Distribution and Bioconcentration of Endocrine Disrupting Chemicals in Surface Water and Fish Bile of the Pearl River Delta, South China. *Chemosphere* **2014**, *107*, 439–446. https://doi.org/10.1016/j.chemosphere.2014.01.048.
(36) Cartner, S.; Eisen, J. S.; Farmer, S. F.; Guillemin, K. J.; Kent, M. L.; Sanders, G. E. *The Zebrafish in Biomedical Research: Biology, Husbandry, Diseases, and Research Applications*; Academic Press, 2019.
(37) Castranova, D.; Lawton, A.; Lawrence, C.; Baumann, D. P.; Best, J.; Coscolla, J.; Doherty, A.; Ramos, J.; Hakkesteeg, J.; Wang, C.; Wilson, C.; Malley, J.; Weinstein, B. M. The Effect of Stocking Densities on Reproductive Performance in Laboratory Zebrafish (Danio Rerio). *Zebrafish* **2011**, *8* (3), 141–146. https://doi.org/10.1089/zeb.2011.0688.
(38) Örn, S.; Andersson, P. L.; Förlin, L.; Tysklind, M.; Norrgren, L. The Impact on Reproduction of an Orally Administered Mixture of Selected PCBs in Zebrafish (Danio Rerio). *Arch. Environ. Contam. Toxicol.* **1998**, *35* (1), 52–57. https://doi.org/10.1007/s002449900348.
(39) Spence, R.; Gerlach, G.; Lawrence, C.; Smith, C. The Behaviour and Ecology of the Zebrafish, Danio Rerio. *Biol. Rev.* **2008**, *83* (1), 13–34. https://doi.org/10.1111/j.1469-185X.2007.00030.x.
(40) Grumetto, L.; Barbato, F.; Russo, G. Scrutinizing the Interactions between Bisphenol Analogues and Plasma Proteins: Insights from Biomimetic Liquid Chromatography, Molecular Docking Simulations and in Silico Predictions. *Environ. Toxicol. Pharmacol.* **2019**, *68*, 148–154. https://doi.org/10.1016/j.etap.2019.02.008.
(41) Edginton, A. N.; Ritter, L. Predicting Plasma Concentrations of Bisphenol A in Children Younger than 2 Years of Age after Typical Feeding Schedules, Using a Physiologically Based Toxicokinetic Model. *Environ. Health Perspect.* **2009**, *117* (4), 645–652. https://doi.org/10.1289/ehp.0800073.
(42) Staples, C. A.; Dome, P. B.; Klecka, G. M.; Oblock, S. T.; Harris, L. R. A Review of the Environmental Fate, Effects, and Exposures of Bisphenol A. *Chemosphere* **1998**, *36* (10), 2149–2173. https://doi.org/10.1016/S0045-6535(97)10133-3.
(43) Fay, K. A.; Fitzsimmons, P. N.; Hoffman, A. D.; Nichols, J. W. Optimizing the Use of Rainbow Trout Hepatocytes for Bioaccumulation Assessments with Fish. *Xenobiotica* **2014**, *44* (4), 345–351. https://doi.org/10.3109/00498254.2013.845704.
(44) Nichols, J.; Fay, K.; Bernhard, M. J.; Bischof, I.; Davis, J.; Halder, M.; Hu, J.; Johanning, K.; Laue, H.; Nabb, D.; Schlechtriem, C.; Segner, H.; Swintek, J.; Weeks, J.; Embry, M. Reliability of In Vitro Methods Used to Measure Intrinsic Clearance of Hydrophobic Organic Chemicals by Rainbow Trout: Results of an International Ring Trial. *Toxicol. Sci.* **2018**, *164* (2), 563–575. https://doi.org/10.1093/toxsci/kfy113.
(45) Nichols, J. W.; Huggett, D. B.; Arnot, J. A.; Fitzsimmons, P. N.; Cowan-Ellsberry, C. E. Toward Improved Models for Predicting Bioconcentration of Well-Metabolized Compounds by Rainbow Trout Using Measured Rates of in Vitro Intrinsic Clearance. *Environ. Toxicol. Chem.* **2013**, *32* (7), 1611–1622. https://doi.org/10.1002/etc.2219.

(46) Li, M.; Yang, Y.; Yang, Y.; Yin, J.; Zhang, J.; Feng, Y.; Shao, B. Biotransformation of Bisphenol AF to Its Major Glucuronide Metabolite Reduces Estrogenic Activity. *PLOS ONE* **2013**, *8* (12), e83170. https://doi.org/10.1371/journal.pone.0083170.
(47) Matthews, J. B.; Twomey, K.; Zacharewski, T. R. In Vitro and in Vivo Interactions of Bisphenol A and Its Metabolite, Bisphenol A Glucuronide, with Estrogen Receptors α and β. *Chem. Res. Toxicol.* **2001**, *14* (2), 149–157. https://doi.org/10.1021/tx0001833.
(48) Sakamoto, H.; Yokota, H.; Kibe, R.; Sayama, Y.; Yuasa, A. Excretion of Bisphenol A-Glucuronide into the Small Intestine and Deconjugation in the Cecum of the Rat. *Biochim. Biophys. Acta BBA - Gen. Subj.* **2002**, *1573* (2), 171–176. https://doi.org/10.1016/S0304-4165(02)00418-X.
(49) Ginsberg, G.; Rice, D. C. Does Rapid Metabolism Ensure Negligible Risk from Bisphenol A? *Environ. Health Perspect.* **2009**, *117* (11), 1639–1643. https://doi.org/10.1289/ehp.0901010.
